# Detecting and distinguishing indicators of risk for suicide using clinical records

**DOI:** 10.1038/s41398-022-02051-4

**Published:** 2022-07-13

**Authors:** Brian K. Ahmedani, Cara E. Cannella, Hsueh-Han Yeh, Joslyn Westphal, Gregory E. Simon, Arne Beck, Rebecca C. Rossom, Frances L. Lynch, Christine Y. Lu, Ashli A. Owen-Smith, Kelsey J. Sala-Hamrick, Cathrine Frank, Esther Akinyemi, Ganj Beebani, Christopher Busuito, Jennifer M. Boggs, Yihe G. Daida, Stephen Waring, Hongsheng Gui, Albert M. Levin

**Affiliations:** 1 Henry Ford Health, Center for Health Policy & Health Services Research, 1 Ford Place, Suite 3A, Detroit, MI 48202 USA; 2 Henry Ford Health, Behavioral Health Services, Detroit, MI USA; 3 Henry Ford Health, Public Health Sciences, Detroit, MI USA; 4 Henry Ford Health, Center for Bioinformatics, Detroit, MI USA; 5 Kaiser Permanente Washington, Health Research Institute, Seattle, WA USA; 6 Kaiser Permanente Colorado, Institute for Health Research, Aurora, CO USA; 7 HealthPartners Institute, Minneapolis, MN USA; 8 Kaiser Permanente Northwest, Center for Health Research, Portland, OR USA; 9 Harvard Pilgrim Health Care Institute & Harvard Medical School, Department of Population Health, Boston, MA USA; 10 Georgia State University & Kaiser Permanente Georgia, Atlanta, GA USA; 11 Kaiser Permanente Hawaii, Center for Integrated Health Care Research, Honolulu, HI USA; 12 Essentia Institute of Rural Health, Duluth, MN USA

**Keywords:** Psychiatric disorders, Scientific community

## Abstract

Health systems are essential for suicide risk detection. Most efforts target people with mental health (MH) diagnoses, but this only represents half of the people who die by suicide. This study seeks to discover and validate health indicators of suicide death among those with, and without, MH diagnoses. This case-control study used statistical modeling with health record data on diagnoses, procedures, and encounters. The study included 3,195 individuals who died by suicide from 2000 to 2015 and 249,092 randomly selected matched controls, who were age 18+ and affiliated with nine Mental Health Research Network affiliated health systems. Of the 202 indicators studied, 170 (84%) were associated with suicide in the discovery cohort, with 148 (86%) of those in the validation cohort. Malignant cancer diagnoses were risk factors for suicide in those without MH diagnoses, and multiple individual psychiatric-related indicators were unique to the MH subgroup. Protective effects across MH-stratified models included diagnoses of benign neoplasms, respiratory infections, and utilization of reproductive services. MH-stratified latent class models validated five subgroups with distinct patterns of indicators in both those with and without MH. The highest risk groups were characterized via high utilization with multiple healthcare concerns in both groups. The lowest risk groups were characterized as predominantly young, female, and high utilizers of preventive services. Healthcare data include many indicators of suicide risk for those with and without MH diagnoses, which may be used to support the identification and understanding of risk as well as targeting of prevention in health systems.

## Introduction

Suicide is a major public health concern. In the United States (US), > 47,000 individuals died by suicide in 2019 [1] – a 25% increase since 2000 [2]. There is an urgent need to develop and implement effective strategies to prevent suicide using timely and accurate data to detect individuals at risk and to inform clinical outreach [3]. According to the most recent US National Strategy for Suicide Prevention, healthcare settings are important for suicide prevention [4–6], where they often target interventions to patients ‘known’ to be at-risk. The first step in the US suicide research strategy is to determine how to best detect individuals at-risk to strategically target suicide prevention [4].

Several innovative statistical models leveraging electronic health records (EHR) or claims data have been developed to better detect suicide risk in health systems [3], but there remain important gaps. First, most US-based studies have examined suicide ideation or attempt outcomes, and there remains an unmet need to understand risk of suicide death. Second, most of the known risk factors associated with suicide are mental health (MH) diagnoses (including MH, substance use disorders, and prior suicide attempts) [7–11]. Nonetheless, recent data show that while over 83% of individuals make a healthcare visit before suicide, 50% do not have a MH diagnosis [12]. Furthermore, approximately 15% of the total US population has a MH diagnosis, and half of all suicides occur among these individuals [13, 14]. However, the other half of all US suicides occur among the 85% of the population without a MH diagnosis [2, 12]. Thus, suicide risk detection and prevention directed primarily towards individuals with a known MH diagnosis can only reach half of all individuals before their death. More information is needed to identify and understand suicide risk among those without known suicide risk factors.

While two-thirds of healthcare visits before suicide occur in outpatient primary care, medical specialty settings, or the emergency department without a documented MH diagnosis [12, 15, 16], there is limited information on non-MH risk factors that may support risk detection [15, 17–20]. Clinical data may provide a solution.

This study fills major gaps by leveraging standard EHR and claims data to detect indicators of suicide risk and protection in the largest case-control study of suicide mortality among individuals receiving care in US health systems to date. Importantly, this study compares models for both those with and without MH diagnoses to juxtapose associated clinical exposures in these groups.

## Methods

This case-control study was conducted within the Mental Health Research Network (MHRN). MHRN is a NIMH-funded consortium of health systems serving 30 million individuals per year. The 9 systems participating in this study are Essentia Health (Minnesota), Harvard Pilgrim Healthcare (Massachusetts), HealthPartners (Minnesota), Henry Ford Health System (Michigan), and the Colorado, Georgia, Hawaii, Northwest, and Washington state regions of Kaiser Permanente. The institutional review boards at each system approved data use for this study.

Cases were defined as individuals who died by suicide from January 1, 2000, through September 30, 2015, and were members of the participating health systems for at least 10 months of the year before death. The 10-month period is selected as individuals are often disenrolled from their health plan during the month of their death. Suicide deaths were identified by ICD-10 codes documented within official State government mortality records, which are the State-level data that comprise the National Death Index [21]. For each case, the date of death was used as a reference date to select matched controls, which were individuals affiliated with the same institution who did not die from suicide during that year. A total of 100 control individuals were randomly selected to match each case. The case-control study included 339,360 individuals—3,360 cases and 336,000 controls. Data were extracted for the 365-day period prior to the reference date for each matched set to focus on near-term indicators. Childhood (<18 years old) cases composed a minority of the sample (n = 165, 4.9%) and were excluded from the analysis. The sample after exclusions was 252,287 adults —3,195 cases and 249,092 controls. Before performing analyses, case-control sets were randomly split into discovery (2/3) and validation (1/3) samples. The sample was also annotated by those with and without a MH diagnosis (defined by those with a past year ICD 9 mental health diagnosis 290–319 or suicide attempt diagnosis E950-E959).

MHRN utilizes a virtual data warehouse to facilitate data sharing across sites [22]. Healthcare indicators for analysis included diagnoses summarized at the ICD-9 sub-chapter level, procedures at the Current Procedural Terminology (CPT) category level, and encounters at the subtype level, including hospitalizations, emergency department visits, and both outpatient visits to primary care and medical specialty settings. Visits to medical specialty settings were subdivided by clinical specialty.

For analysis, each healthcare indicator was treated as binary (“ever” = 1 or “never” = 0). Those indicators with less than 10 observations were excluded from subsequent analyses. All data collected at day 0 (the reference day), as well as emergency encounters on day 1 were excluded to avoid including data from the actual suicide. After exclusions, the analysis included 107 diagnosis sub-chapters, 67 procedure categories, and 28 encounter types.

### Statistical Analysis

Supplementary Fig. 1 displays the analytical goals of the study (identification of 1. healthcare indicators associated with suicide and 2) sub-groups of at-risk patients) in the context of the methods and the corresponding sample sizes. To account for the matched design, conditional logistic regression models were used to test associations between suicide and indicators. All models were adjusted for age and sex. Differential effects by MH status were evaluated through the inclusion of a multiplicative interaction term between MH diagnosis and each indicator, with significance assessed using a likelihood ratio test. To account for multiple testing, indicators with false discovery rate adjusted p-values (FDR) < 0.05 were considered significant in the discovery sample, and those with unadjusted p-values <0.05 in the validation sample were considered validated.

Following single indicator analyses that demonstrated heterogeneity by MH status, two approaches were taken to assess multi-indicator associations with suicide. First, separate multi-indicator models were constructed for those with and without a MH diagnosis using conditional logistic models fit with a lasso penalty. These analyses were conducted using the R package *clogitL1* [23], with the lasso shrinkage parameter (λ) determined using 10-fold cross-validation. For each stratum, discovery and validation models were fit using all indicators. For those indicators retained in both models, a standard conditional logistic regression model was fit to calculate 95% confidence intervals (95% CI) and tests for statistical significance in the full sample. The second approach was patient-centric, utilizing latent class analysis (LCA) to determine the existence of distinct risk sub-groups. LCA was stratified by MH status using the indicators retained in both the stratum-specific discovery and validation multi-indicator penalized models. For each LCA model, the number of classes was determined based on the Bayesian information criterion (BIC) and class interpretability in the corresponding discovery samples, and confirmatory LCA was performed to assess consistency of case percentages and utilization profiles in the corresponding validation samples. LCA was performed using MPlus v8.3. All other analyses were performed using R v3.6.1. The program and statistical code, data codebook, and dataset are available upon request and approval by the study team.

## Results

### Study sample description

Summary statistics for demographic variables are shown in Table 1. Overall, cases were older (mean age for cases was 51.4 years and 48.1 years for controls) and more likely to be male (cases 77.5% and controls 46.2%). Cases were less likely to have commercial insurance (cases 62.8% and controls 72.5%), more likely to live in areas with lower education levels (cases 39.1% and controls 36.9%), and had lower neighborhood household incomes (case median = $65,567 and control median = $67,694). Table 1 also shows that case/control summary statistics were consistent between discovery and validation samples.

**Table 1:** Demographic characteristics of study subjects by suicide case/control status.

	All			Discovery			Validation		
	Case (*n* = 3195)	Control (*n* = 249,092)	*p* value*	Case (*n* = 2130)	Control (*n* = 166,076)	*p* value*	Case (*n* = 1065)	Control (*n* = 83,016)	*p* value*
Age, mean (SD)	51.44 (18.20)	48.13 (17.19)	<0.001	51.31 (18.26)	48.13 (17.16)	<0.001	51.71 (18.08)	48.13 (17.25)	<0.001
Sex, *n* (%)			<0.001			<0.001			<0.001
Male	2,476 (77.5)	115,097 (46.2)		1662 (78.0)	76,635 (46.1)		814 (76.4)	38,462 (46.3)	
Female	719 (22.5)	133,995 (53.8)		468 (22.0)	89,441 (53.9)		251 (23.6)	44,554 (53.7)	
Lower Education, n (%)			0.030			0.014			0.852
Yes	1250 (39.1)	91,960 (36.9)		846 (39.7)	61,110 (36.8)		404 (37.9)	30,850 (37.2)	
No	1852 (58.0)	150,102 (60.3)		1218 (57.2)	100,161 (60.3)		634 (59.5)	49,941 (60.2)	
Insurance, n (%)			<0.001			<0.001			<0.001
Commercial	2005 (62.8)	180,508 (72.5)		1332 (62.5)	120,390 (72.5)		673 (63.2)	60,118 (72.4)	
Public	830 (26.0)	42,333 (17.0)		561 (26.3)	28,063 (16.9)		269 (25.3)	14,270 (17.2)	
Other	360 (11.3)	26,251 (10.5)		237 (11.1)	17,623 (10.6)		123 (11.5)	8,628 (10.4)	
Median Household Income, mean (SD)	65,567 (28,203)	67,694 (28,572)	<0.001	65,151 (28,039)	67,687 (28,490)	<0.001	66,394 (28,521)	67,709 (28,736)	0.143

Abbreviations: *n* indicates number of individuals, *%*, column percent, *SD* standard deviation.

*P values correspond to chi-squared tests for categorical variables and t-tests for continuous variables.

### Single healthcare indicator associations with suicide death and heterogeneity by MH status

The single indicator association results are included in Supplementary Table 1. Overall, of the 202 indicators tested in the discovery sample, 170 were significantly associated with suicide death (FDR < 0.05), and of these, 146 (86%) were also significant in the validation sample, including 78 diagnoses (77 increased/1 decreased risk), 45 procedures (44/1), and 21 encounter types (21/0). The top ten most significant, validated associations for each indicator type are summarized in Table 2.

**Table 2:** Univariate odds ratios for death by suicide adjusted by age and sex.

	Discovery			Validation		
	OR (95% CI)	*p* value*	FDR-adjusted *p*-value*	OR (95% CI)	*p* value*	FDR-adjusted *p*-value*
Diagnosis Sub-Chapter						
Poisoning by Drugs, Medicinal and Biological Substances	47.84 (38.52–59.40)	<0.001	<0.001	43.70 (32.18–59.35)	<0.001	<0.001
Other Psychoses	10.41 (9.45–11.46)	<0.001	<0.001	9.96 (8.69–11.42)	<0.001	<0.001
Neurotic Disorders, Personality Disorders, And Other Nonpsychotic Mental Disorders	5.91 (5.41–6.45)	<0.001	<0.001	6.21 (5.48–7.03)	<0.001	<0.001
Organic Psychotic Conditions	5.65 (4.75–6.73)	<0.001	<0.001	6.39 (5.08–8.04)	<0.001	<0.001
Pain	4.44 (3.71–5.31)	<0.001	<0.001	4.15 (3.20–5.38)	<0.001	<0.001
Other Diseases of Respiratory System	4.23 (3.67–4.87)	<0.001	<0.001	4.55 (3.75–5.52)	<0.001	<0.001
Other Disorders of The Central Nervous System	4.13 (3.59–4.76)	<0.001	<0.001	4.04 (3.31–4.94)	<0.001	<0.001
Persons Encountering Health Services in Other Circumstances	2.99 (2.70–3.31)	<0.001	<0.001	2.97 (2.56–3.43)	<0.001	<0.001
Persons Encountering Health Services for Specific Procedures and Aftercare	2.54 (2.28–2.82)	<0.001	<0.001	2.24 (1.92–2.60)	<0.001	<0.001
Symptoms	2.52 (2.30–2.76)	<0.001	<0.001	2.58 (2.27–2.93)	<0.001	<0.001
Encounter Type						
Nonacute Institutional Stay - Rehab	8.82 (5.85–13.32)	<0.001	<0.001	7.40 (4.07–13.45)	<0.001	<0.001
Acute Inpatient - Acute Inpatient	6.51 (5.87–7.22)	<0.001	<0.001	5.93 (5.11–6.88)	<0.001	<0.001
Nonacute Institutional Stay - Skilled Nursing	4.08 (2.98–5.58)	<0.001	<0.001	3.32 (2.11–5.23)	<0.001	<0.001
Emergency - Hospital Ambulatory	4.00 (3.64–4.39)	<0.001	<0.001	4.00 (3.50–4.57)	<0.001	<0.001
Ambulatory - Observation Bed	3.30 (2.59–4.20)	<0.001	<0.001	2.25 (1.51–3.36)	<0.001	<0.001
Other Nonovernight - Home Health	2.59 (2.21–3.02)	<0.001	<0.001	2.50 (2.00–3.13)	<0.001	<0.001
Telephone - Other Nonhospital	2.25 (1.97–2.56)	<0.001	<0.001	2.18 (1.80–2.63)	<0.001	<0.001
Ambulatory - Hospital Ambulatory	1.85 (1.66–2.06)	<0.001	<0.001	1.85 (1.59–2.16)	<0.001	<0.001
Ambulatory - Outpatient Clinic	1.81 (1.58–2.07)	<0.001	<0.001	1.63 (1.36–1.97)	<0.001	<0.001
Other Nonovernight - Other Nonhospital	1.80 (1.57–2.06)	<0.001	<0.001	1.44 (1.17–1.76)	<0.001	<0.001
Procedure Type						
Critical Care Services	20.35 (17.41–23.79)	<0.001	<0.001	19.69 (15.73–24.65)	<0.001	<0.001
Drug Testing	13.17 (11.35–15.29)	<0.001	<0.001	15.12 (12.21–18.71)	<0.001	<0.001
Psychiatry	7.92 (7.17–8.74)	<0.001	<0.001	8.04 (6.99–9.25)	<0.001	<0.001
Hospital Inpatient Services	7.71 (6.85–8.67)	<0.001	<0.001	7.61 (6.44–8.99)	<0.001	<0.001
Therapeutic Drug Assays	6.59 (5.69–7.64)	<0.001	<0.001	7.36 (6.01–9.02)	<0.001	<0.001
Respiratory System	5.59 (4.64–6.73)	<0.001	<0.001	6.42 (5.06–8.14)	<0.001	<0.001
Emergency Department Services	4.65 (4.24–5.11)	<0.001	<0.001	5.10 (4.48–5.80)	<0.001	<0.001
Hydration, Therapeutic, Prophylactic, Diagnostic Injections and Infusions, and Chemotherapy and Other Highly Complex Drug or Highly Complex Biologic Agent Administration	3.45 (2.99–3.96)	<0.001	<0.001	3.20 (2.62–3.91)	<0.001	<0.001
Cardiovascular	3.06 (2.78–3.37)	<0.001	<0.001	2.92 (2.55–3.33)	<0.001	<0.001
Hematology and Coagulation	2.25 (2.06–2.47)	<0.001	<0.001	2.37 (2.09–2.70)	<0.001	<0.001

Abbreviations: *OR* indicates odds ratio, *95% CI* 95% confidence interval, *n* number of individuals.

*Likelihood ratio test *p* value from a conditional logistic regression adjusted for age and sex.

All indicators were evaluated for differential suicide risk by MH status (Supplementary Table 2). Of the 202 individual healthcare indicators, 44 had significant interactions (FDR < 0.05) with MH status in the discovery set, with 7 (16%) also significant in the validation (*p* < 0.05), with MH-stratified results displayed in Table 3. Of note, malignant neoplasms were associated with >twofold higher risk among those without MH diagnoses. Given these differences, subsequent multi-indicator analyses were stratified by MH status.

**Table 3:** Mental health stratified odds ratios for validated interactions between utilization features and mental health status.

	Discovery					Validation				
	Mental Health		Non-Mental Health			Mental Health		Non-Mental Health		
	OR (95% CI)	*p* value*	OR (95% CI)	*p* value*	Interaction *p* value*	OR (95% CI)	*p* value*	OR (95% CI)	*p* value*	Interaction *p* value^†^
Diagnosis Sub-Chapter										
Malignant Neoplasms of Other and Unspecified Sites	2.08 (1.42–3.07)	<0.001	6.78 (4.61–9.97)	<0.001	<0.001	1.48 (0.83–2.63)	0.179	7.69 (4.35–13.58)	<0.001	<0.001
Persons Encountering Health										
Services in Other Circumstances	2.24 (1.97–2.54)	<0.001	1.26 (1.01–1.58)	0.043	0.001	2.23 (1.87–2.67)	<0.001	1.24 (0.89–1.72)	0.205	0.009
Malignant Neoplasms of Respiratory and Intrathoracic Organs	2.50 (1.43–4.38)	0.001	7.17 (3.89–13.22)	<0.001	0.002	0.87 (0.30–2.47)	0.791	8.28 (3.78–18.14)	<0.001	<0.001
Encounter Type										
Emergency - Hospital Ambulatory	3.06 (2.71–3.45)	<0.001	1.65 (1.34–2.03)	<0.001	<0.001	3.06 (2.58–3.62)	<0.001	1.65 (1.23–2.21)	0.001	<0.001
Other Nonovernight - Home Health	1.40 (1.15–1.71)	0.001	2.70 (2.05–3.57)	<0.001	<0.001	1.30 (0.99–1.71)	0.060	2.57 (1.68–3.94)	<0.001	0.008
Nonacute Institutional Stay - Skilled Nursing	1.82 (1.25–2.65)	0.002	5.38 (2.80–10.36)	<0.001	<0.001	1.10 (0.64–1.92)	0.724	6.46 (2.64–15.81)	<0.001	<0.001
Procedure Type										
Emergency Department Services	3.41 (3.02–3.84)	<0.001	2.20 (1.81–2.66)	<0.001	<0.001	3.88 (3.28–4.59)	<0.001	2.24 (1.71–2.93)	<0.001	0.001

Abbreviations: *OR* indicates odds ratio, *95% CI* 95% confidence interval.

^*^Likelihood ratio test p-value from a conditional logistic regression adjusted for age and sex.

†Likelihood ratio test p-value for the multiplicative interaction effect of MH status-by-health care metric assessed in a conditional logistic regression adjusted for age and sex.

### Multi-indicator associations with suicide by MH status

The results from the discovery/validation penalized conditional logistic regression models stratified by MH status are displayed in Supplementary Table 3. The MH model retained 87 indicators (46 diagnoses, 30 procedures, and 11 encounters), and in the validation model, 49 indicators (24 diagnoses, 19 procedures, and 6 encounters) were retained. Increased/decreased risk of suicide was significantly associated with 7/8 diagnoses, 9/2 procedures, and 2/0 encounters. The non-MH discovery model retained 98 indicators (56 diagnoses, 29 procedures, and 13 encounters), and in the corresponding validation model, 51 indicators (25 diagnoses, 8 procedures, and 18 encounters) were retained. Increased/decreased risk was significantly associated with 7/4 diagnoses, 5/4 procedures, and 2/2 encounters.

**Fig. 1:**
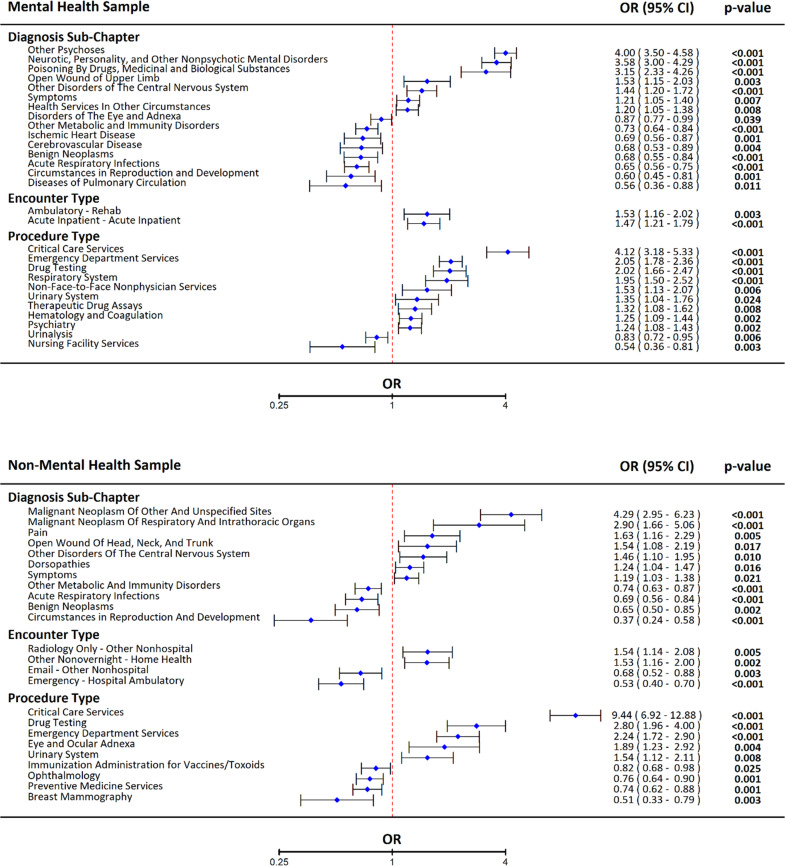
Forest plots of odds ratios from multi-indicator models of suicide death among those A) with and B) without a mental health diagnosis. Results in both panels are taken from conditional logistic regression models fit to the full sample of those individual with and without mental health diagnoses. For each model, indicators were selected from those that were retained in both the discovery and validation penalized regression models, and only those that were statistically significant (*p* < 0.05) in the full sample were included in these figures.

The full MH and non-MH samples were used to estimate 95% CIs and significance for indicators retained in the respective validation models, and the results for those indicators that remained statistically significant are displayed in Fig. 1. For diagnoses, several individual MH conditions increased risk, including Other Psychoses (OR = 4.00, 95% CI 3.50–4.58) and Personality Disorders and Other Nonpsychotic Mental Disorders (OR = 3.58, 95% CI 3.00–4.29). In comparison, cancer diagnoses had a unique increased risk among non-MH individuals, including Malignant Neoplasms of Other and Unspecified Sites (OR = 4.29, 95% CI 2.95–6.23) and Malignant Neoplasm of Respiratory and Intrathoracic Organs (OR = 2.90, 95% CI 1.66–5.06). In contrast, Benign Neoplasms had protective effects for both non-MH (OR = 0.65, 95% CI 0.50–0.85) and MH (OR = 0.68, 95% CI 0.55–0.84) individuals. The services related to Reproduction and Development were protective in both models, although the effect was more extreme in the non-MH (OR = 0.37, 95% CI 0.24–0.58) relative to the MH (OR = 0.60, 95% CI 0.45–0.81) model.

For procedures, increased risk among MH individuals was distinguished by procedures related to the Respiratory System (OR = 1.95, 95% CI 1.50–2.52) and Psychiatry (OR = 1.24, 95% CI 1.08–1.43). In comparison, Radiology procedures (OR = 1.54, 95% CI 1.14–2.08) were significant for the non-MH model. The MH sample had a unique protective effect of Nursing Facility Services (OR = 0.54, 95% CI 0.36–0.81). Non-MH individuals had unique protective effects of Immunization Administration for Vaccines/Toxoids (OR = 0.82, 95% CI 0.68–0.98), Preventive Medicine Services (OR = 0.74, 95% CI 0.62–0.88), and Ophthalmology (OR = 0.76, 95% CI 0.64–0.90).

For encounters, increased risk among MH individuals was distinguished by Non-Face-to-Face Non-physician Services (OR = 1.53, 95% CI 1.13–2.07), Acute Inpatient (OR = 1.47, 95% CI 1.21–1.79), and Ambulatory - Rehab (OR = 1.53, 95% CI 1.16–2.02). For non-MH individuals, Other Non-overnight - Home Health (OR = 1.53, 95% CI 1.16–2.00) and Radiology Only - Outpatient Clinic (OR = 1.54, 95% CI 1.14–2.08) were associated with increased risk. MH individuals had no encounter types with protective effects, and non-MH individuals had protective effects for Emergency - Hospital Ambulatory (OR = 0.53, 95% CI 0.40–0.70) and Email - Other Non-hospital (OR = 0.68, 95% CI 0.52–0.88).

### Latent class analysis discovery and validation of distinct patient suicide risk sub-groups

**Fig. 2:**
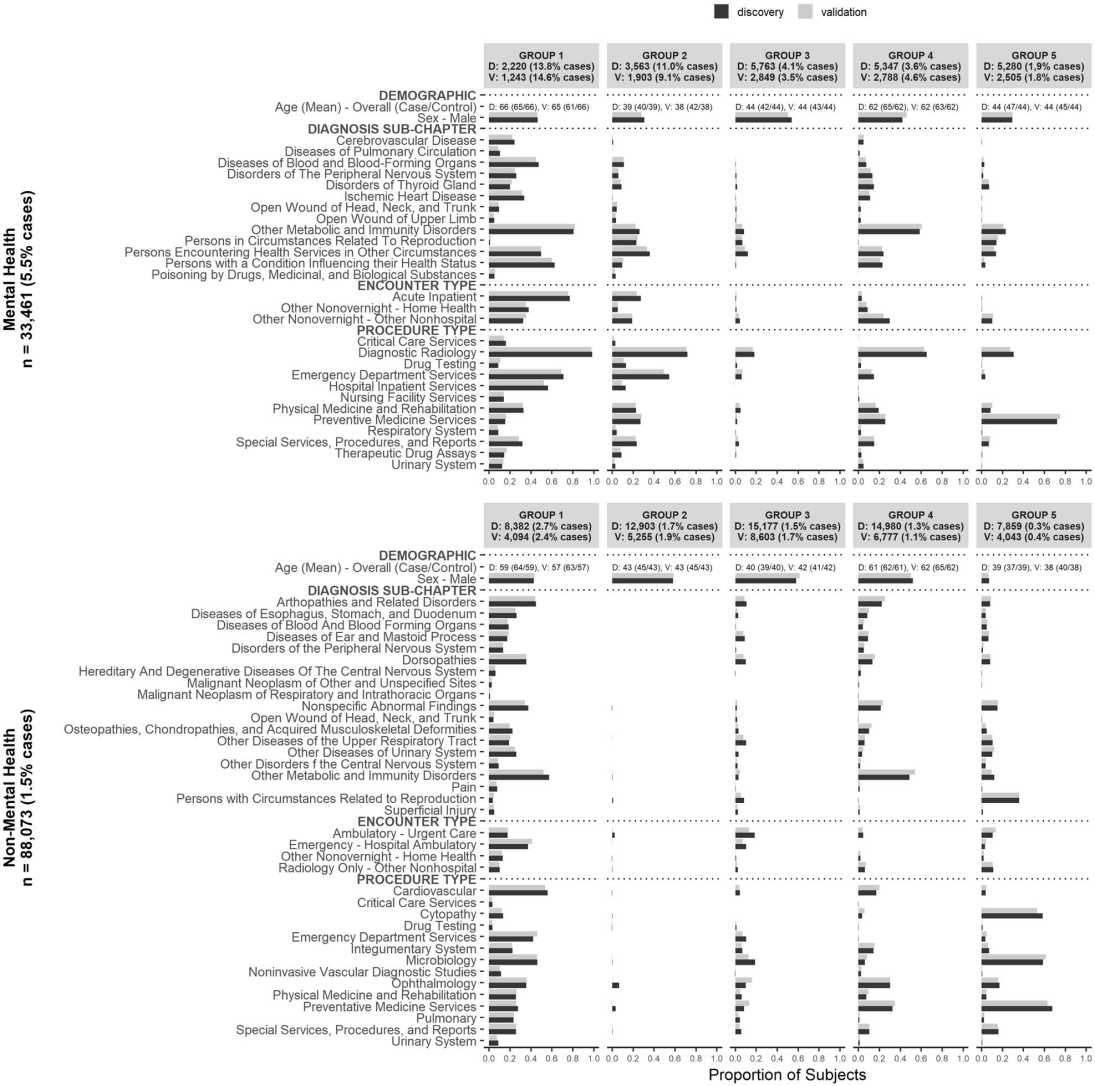
Latent class analysis sub-group identification based on health care indicators associated with suicide death in individuals A) with and B) without a mental health diagnosis. For each mental health stratum, latent class analysis (LCA) was performed based on health care indicators identified in the respective penalized regression models in the discovery sample. LCA was performed in the discovery sample (D), followed by confirmatory LCA in the validation sample (V). The frequency of cases is displayed overall and for each LCA sub-group for both D and V samples. Within each stratum, the LCA sub-group-specific frequency are displayed for those health care indicators where at least 9 of the 10 ratios of the pairwise group frequencies were >1.5 (ie. sub-group distinguishing indicators).

The top indicators that differentiate the LCA identified sub-groups for both MH and non-MH samples are displayed in Fig. 2. For both the MH and non-MH samples, the number of latent subgroups was identified as five, based on lowest value past the inflection point in the BIC curve (Supplementary Fig. 2) that also identified a low-risk group. For the MH sample, the groups are labelled in order of decreasing case percentage: Group 1 (10.0% of the sample, 13.8% cases), Group 2 (16.1% of the sample, 11.0% cases), Group 3 (26.0% of the sample, 4.1% cases), Group 4 (24.1% of the sample, 3.6% cases), and Group 5 (23.8% of the sample, 1.9% cases). Based on these values, Groups 1 and 2 were identified as high-risk groups for suicide. Specifically, Group 1 had higher proportions of many diagnosis sub-chapters, procedure types, and encounter types, signifying a high utilization group with multiple healthcare concerns. Group 2 had a similar but less extreme profile, and these individuals were also younger (39 years) and more likely to be female. Group 5 had the lowest case prevalence. This group was one of the youngest on average (44 years old), had the lowest proportion of males, and the highest proportion of routine/preventive health visits. Confirmatory LCA of the five-group solution in the validation sample yielded groups with similar case percentages and healthcare indicator profiles (Fig. 3).

For non-MH individuals, he resulting groups and their corresponding case proportions were: Group 1 (14.1% of the sample, 2.7% cases), Group 2 (21.8% of the sample, 1.7% cases), Group 3 (25.6% of the sample, 1.5% cases), Group 4 (25.3% of the sample, 1.3% cases), and Group 5 (13.3% of the sample, 0.3% cases). The noticeably high-risk Group 1 was the second oldest (59 years old) and contained much higher percentages of most healthcare indicators with multiple concerns, similar to Groups 1 and 2 from the MH sample. Further, while Groups 3 and 4 where at intermediate risk and had similar but less extreme healthcare profiles, there was an additional disengaged, high risk group (Group 2) unique to the non-MH sample. The lowest-risk Group 5 was the youngest (39 years), contained a low proportion of males (5.6%), and displayed higher proportions of routine/preventive visits – similar to Group 5 from the MH model. Confirmatory LCA of the five-group solution resulted in both similar case proportions and healthcare indicator profiles.

## Discussion

Clinical data can be used to better differentiate the structure of suicide risk as well as to identify subgroups and service settings that require additional attention for suicide prevention in health systems. Importantly, this study employed multiple methods to identify and validate individual clinical indicators and patterns of those indicators, to better distinguish risk in not only those with MH diagnoses, but also among those with previously unknown risk factors. The insights into these groups with respective high and low population suicide incidence can inform clinical outreach and assessment strategies, and as such, this study adds multiple clinically and methodologically significant findings to the literature. The findings from the multi-indicator models and LCA may be most relevant and can help inform future work to determine how these complex indicators of risk can inform clinical outreach.

First, this study confirms prior research indicating that those who die by suicide often have multiple chronic or complex conditions [17, 24]. In both the MH and non-MH analyses, individuals with the highest healthcare utilization had the greatest risk for suicide, indicating an opportunity for intervention. Prior studies have focused analyses on single clinical risk factors for suicide [19, 25–27], but this study suggests that future research should also consider comorbidities to better distinguish risk. Suicide prevention in health systems should focus on those individuals who are frequent utilizers of services, potentially indicating higher severity of multiple conditions.

Second, older individuals with MH conditions who have low utilization may be at elevated risk. In contrast, younger females who are more engaged in routine healthcare are at lower risk. The Interpersonal Theory of Suicide indicates that isolation and burdensomeness may be important indicators of suicide [28]. These findings collectively indicate that engagement or connectedness with health systems, particularly for regular routine care, may be protective. This emphasizes the importance of clinical or community outreach in suicide prevention, especially for older individuals or males who have become disconnected from care.

Third, while suicide prevention has traditionally focused on individuals with MH conditions, we found many non-MH medical indicators associated with suicide. This supports the importance of ensuring use of low-intensity suicide risk detection methods such as the implementation of EHR-based statistical models or routine clinical screening and assessment [3, 29, 30] in primary care and other non-behavioral health settings. Brief interventions, such as safety or crisis response planning [31, 32], can be immediately offered to those at higher risk in addition to the connection with appropriate levels of follow-up specialty care.

Fourth, there were several novel protective factors for suicide identified in the study. Past studies have found few clinical protective factors for suicide. Benign cancer diagnoses were associated with reduced risk and may indicate a positive life event that contrasts other life stressors. Receipt of preventive services and vaccinations, also associated with lower risk, may suggest that those more engaged in overall health promotion and prevention have lower risk. These factors deserve more in-depth study in future research.

Fifth, the modeling plan implemented in this study was particularly innovative. Risk detection models require prespecification of exposure variables. Thus, recent predictive modeling studies have focused primarily on behavioral health factors as primary exposures [33–37]. MH conditions were the primary factors used in risk detection in prior studies. This was important and valuable given that health systems, including the Veterans Health Administration, have begun implementing these models to stimulate clinical action [3, 38]. However, the current study uniquely identified non-MH clinical risk profiles that can help detect risk among those without MH diagnoses and differentiate risk among those with MH risk factors. The new models developed and validated in this study can also provide guidance to improve future models, including non-MH indicators.

These findings must be viewed in the context of limitations. First, the study was conducted in large health systems among individuals with health insurance. While the results are generalizable to those with many insurance types (public and commercial), individuals without insurance were not included. Second, this study used ICD-9 diagnosis codes. Future studies should replicate findings using ICD-10 codes recently implemented in practice. Third, this study used a case-control design, which is efficient for the estimation of indictor effects. Future studies may leverage these findings to inform absolute risk estimates using prospective cohorts. Fourth, race/ethnicity was not included, as it was not ascertained by health systems prior to 2009. While the participating systems are diverse, this may be important to include in future models. Fifth, while the validation hold-out sample did not overlap with those in the discovery, it was not possible to perform the confirmatory LCA in a separate set of health care systems. Finally, all healthcare utilization occurring on the date of, or day prior to, death were excluded to account for potential utilization that may have been part of the suicide death.

Clinical data include a range of health indicators, both risk and protective, which can be used to better detect suicide risk among those without known MH diagnoses and to better distinguish risk among those with MH diagnoses. These data can also inform targeted clinical outreach, assessment, and follow-up to those at highest risk. This may be particularly important for those with multiple conditions and those disengaged from healthcare. Suicide prevention efforts should span across the entire health system.

## Supplementary information

Supplementary Tables

Supplementary Figure 1

Supplementary Figure 2
