# Supplementary material for: Detecting and distinguishing indicators of risk for suicide using clinical records
Source: Transl Psychiatry. 2022 Jul 13;12:280. doi: 10.1038/s41398-022-02051-4 (PMC9279332; doi:10.1038/s41398-022-02051-4)
Supplement: Supplementary file 1 — Supplementary Tables [file 41398_2022_2051_MOESM1_ESM.docx]

**Supplemental Table 1A.** Single healthcare indicator odds ratios for death by suicide based on ICD-9 diagnosis sub-chapter adjusted by age and sex.

|  | **Discovery**  **(n = 168,206)** | | | |  | **Validation**  **(n = 84,081)** | | | |
| --- | --- | --- | --- | --- | --- | --- | --- | --- | --- |
| **Diagnosis Sub-Chapter** | **OR** | **95% CI** | **p-value** | **FDR** |  | **OR** | **95% CI** | **p-value** | **FDR** |
| Poisoning by Drugs, Medicinal and Biological Substances | 47.84 | 38.52-59.40 | <0.001 | <0.001 |  | 43.70 | 32.18-59.35 | <0.001 | <0.001 |
| Internal Injury of Thorax, Abdomen, And Pelvis | 12.03 | 7.61-19.02 | <0.001 | <0.001 |  | 6.11 | 2.77-13.46 | <0.001 | <0.001 |
| Other Psychoses | 10.41 | 9.45-11.46 | <0.001 | <0.001 |  | 9.96 | 8.69-11.42 | <0.001 | <0.001 |
| Fracture of Skull | 9.25 | 6.38-13.42 | <0.001 | <0.001 |  | 14.60 | 8.66-24.60 | <0.001 | <0.001 |
| Pneumoconioses And Other Lung Diseases Due to External Agents | 9.19 | 6.40-13.20 | <0.001 | <0.001 |  | 9.68 | 6.11-15.34 | <0.001 | <0.001 |
| Inflammatory Diseases of The Central Nervous System | 8.57 | 4.38-16.78 | <0.001 | <0.001 |  | 7.27 | 2.56-20.60 | <0.001 | <0.001 |
| Toxic Effects of Substances Chiefly Nonmedicinal as To Source | 7.79 | 5.62-10.80 | <0.001 | <0.001 |  | 9.13 | 5.80-14.37 | <0.001 | <0.001 |
| Intracranial Injury, Excluding Those with Skull Fracture | 7.61 | 5.80-9.99 | <0.001 | <0.001 |  | 7.70 | 5.34-11.10 | <0.001 | <0.001 |
| Neurotic Disorders, Personality Disorders, And Other Nonpsychotic  Mental Disorders | 5.91 | 5.41-6.45 | <0.001 | <0.001 |  | 6.21 | 5.48-7.03 | <0.001 | <0.001 |
| Organic Psychotic Conditions | 5.65 | 4.75-6.73 | <0.001 | <0.001 |  | 6.39 | 5.08-8.04 | <0.001 | <0.001 |
| Malignant Neoplasm of Respiratory and Intrathoracic Organs | 5.18 | 3.50-7.65 | <0.001 | <0.001 |  | 3.87 | 2.13-7.03 | <0.001 | <0.001 |
| Malignant Neoplasm of Lip, Oral Cavity, And Pharynx | 4.79 | 2.40-9.56 | <0.001 | <0.001 |  | 4.45 | 1.78-11.17 | 0.001 | 0.002 |
| Malignant Neoplasm of Other and Unspecified Sites | 4.54 | 3.50-5.88 | <0.001 | <0.001 |  | 3.75 | 2.55-5.51 | <0.001 | <0.001 |
| Pain | 4.44 | 3.71-5.31 | <0.001 | <0.001 |  | 4.15 | 3.20-5.38 | <0.001 | <0.001 |
| Fracture of Neck and Trunk | 4.34 | 3.22-5.86 | <0.001 | <0.001 |  | 3.54 | 2.28-5.49 | <0.001 | <0.001 |
| Other Diseases of Respiratory System | 4.23 | 3.67-4.87 | <0.001 | <0.001 |  | 4.55 | 3.75-5.52 | <0.001 | <0.001 |
| Other Disorders of The Central Nervous System | 4.13 | 3.59-4.76 | <0.001 | <0.001 |  | 4.04 | 3.31-4.94 | <0.001 | <0.001 |
| Late Effects of Injuries, Poisonings, Toxic Effects, And Other External  Causes | 3.80 | 2.52-5.74 | <0.001 | <0.001 |  | 4.79 | 2.81-8.18 | <0.001 | <0.001 |
| Ill-Defined and Unknown Causes of Morbidity and Mortality | 3.76 | 3.18-4.45 | <0.001 | <0.001 |  | 4.11 | 3.26-5.18 | <0.001 | <0.001 |
| Burns | 3.74 | 2.40-5.83 | <0.001 | <0.001 |  | 4.00 | 2.10-7.62 | <0.001 | <0.001 |
| Injury to Nerves and Spinal Cord | 3.51 | 1.85-6.69 | <0.001 | <0.001 |  | 4.45 | 1.93-10.24 | <0.001 | 0.001 |
| Syphilis and Other Venereal Diseases | 3.43 | 1.75-6.72 | <0.001 | <0.001 |  | 0.73 | 0.10-5.24 | 0.752 | 0.808 |
| Hereditary and Degenerative Diseases of The Central Nervous System | 3.28 | 2.73-3.94 | <0.001 | <0.001 |  | 3.32 | 2.57-4.29 | <0.001 | <0.001 |
| Malignant Neoplasm of Digestive Organs and Peritoneum | 3.25 | 2.24-4.73 | <0.001 | <0.001 |  | 3.17 | 1.90-5.29 | <0.001 | <0.001 |
| Open Wound of Head, Neck, And Trunk | 3.15 | 2.57-3.87 | <0.001 | <0.001 |  | 3.72 | 2.85-4.87 | <0.001 | <0.001 |
| Other Diseases of Digestive System | 3.05 | 2.62-3.54 | <0.001 | <0.001 |  | 2.69 | 2.15-3.36 | <0.001 | <0.001 |
| Persons Encountering Health Services in Other Circumstances | 2.99 | 2.70-3.31 | <0.001 | <0.001 |  | 2.97 | 2.56-3.43 | <0.001 | <0.001 |
| Certain Traumatic Complications and Unspecified Injuries | 2.93 | 2.51-3.43 | <0.001 | <0.001 |  | 3.71 | 3.03-4.53 | <0.001 | <0.001 |
| Complications of Surgical and Medical Care, Not Elsewhere Classified | 2.84 | 2.30-3.50 | <0.001 | <0.001 |  | 2.67 | 1.96-3.63 | <0.001 | <0.001 |
| Other and Unspecified Effects of External Causes | 2.83 | 2.31-3.46 | <0.001 | <0.001 |  | 2.95 | 2.24-3.89 | <0.001 | <0.001 |
| Open Wound of Upper Limb | 2.81 | 2.24-3.54 | <0.001 | <0.001 |  | 3.05 | 2.21-4.20 | <0.001 | <0.001 |
| Effects of Foreign Body Entering Through Orifice | 2.77 | 1.92-3.99 | <0.001 | <0.001 |  | 1.06 | 0.47-2.39 | 0.881 | 0.917 |
| Open Wound of Lower Limb | 2.69 | 1.95-3.71 | <0.001 | <0.001 |  | 2.25 | 1.38-3.67 | 0.001 | 0.002 |
| Cerebrovascular Disease | 2.61 | 2.15-3.17 | <0.001 | <0.001 |  | 2.28 | 1.72-3.03 | <0.001 | <0.001 |
| Other Bacterial Diseases | 2.58 | 2.08-3.21 | <0.001 | <0.001 |  | 2.09 | 1.50-2.93 | <0.001 | <0.001 |
| Diseases of The Blood and Blood-Forming Organs | 2.58 | 2.25-2.96 | <0.001 | <0.001 |  | 2.98 | 2.48-3.57 | <0.001 | <0.001 |
| Fracture of Upper Limb | 2.58 | 1.96-3.40 | <0.001 | <0.001 |  | 1.92 | 1.24-2.98 | 0.003 | 0.005 |
| Persons Encountering Health Services for Specific Procedures and  Aftercare | 2.54 | 2.28-2.82 | <0.001 | <0.001 |  | 2.24 | 1.92-2.60 | <0.001 | <0.001 |
| Symptoms | 2.52 | 2.30-2.76 | <0.001 | <0.001 |  | 2.58 | 2.27-2.93 | <0.001 | <0.001 |
| Acquired Absence of Other Organs and Tissue | 2.50 | 1.32-4.72 | 0.005 | 0.006 |  | 1.53 | 0.49-4.84 | 0.466 | 0.526 |
| Nutritional Deficiencies | 2.45 | 2.01-3.00 | <0.001 | <0.001 |  | 2.25 | 1.70-2.98 | <0.001 | <0.001 |
| Other Forms of Heart Disease | 2.44 | 2.14-2.76 | <0.001 | <0.001 |  | 2.22 | 1.85-2.66 | <0.001 | <0.001 |
| Pneumonia and Influenza | 2.38 | 1.97-2.88 | <0.001 | <0.001 |  | 2.43 | 1.88-3.16 | <0.001 | <0.001 |
| Chronic Rheumatic Heart Disease | 2.29 | 1.51-3.46 | <0.001 | <0.001 |  | 1.80 | 0.92-3.52 | 0.086 | 0.107 |
| Rickettsioses and Other Arthropod-Borne Diseases | 2.28 | 1.00-5.19 | 0.050 | 0.060 |  | 3.32 | 1.20-9.18 | 0.021 | 0.028 |
| Fracture of Lower Limb | 2.27 | 1.71-3.01 | <0.001 | <0.001 |  | 2.52 | 1.75-3.64 | <0.001 | <0.001 |
| Diseases of Pulmonary Circulation | 2.09 | 1.48-2.95 | <0.001 | <0.001 |  | 2.08 | 1.29-3.36 | 0.003 | 0.004 |
| Disorders of The Peripheral Nervous System | 2.08 | 1.78-2.42 | <0.001 | <0.001 |  | 1.70 | 1.35-2.15 | <0.001 | <0.001 |
| Diseases of Veins and Lymphatics, And Other Diseases of Circulatory  System | 2.06 | 1.79-2.38 | <0.001 | <0.001 |  | 2.42 | 2.00-2.92 | <0.001 | <0.001 |
| Other Diseases of Intestines and Peritoneum | 2.01 | 1.76-2.30 | <0.001 | <0.001 |  | 2.03 | 1.68-2.46 | <0.001 | <0.001 |
| Nonspecific Abnormal Findings | 1.99 | 1.79-2.20 | <0.001 | <0.001 |  | 1.66 | 1.43-1.93 | <0.001 | <0.001 |
| Diseases of Arteries, Arterioles, And Capillaries | 1.96 | 1.61-2.37 | <0.001 | <0.001 |  | 1.94 | 1.48-2.54 | <0.001 | <0.001 |
| Malignant Neoplasm of Lymphatic and Hematopoietic Tissue | 1.95 | 1.24-3.05 | 0.004 | 0.005 |  | 2.73 | 1.64-4.56 | <0.001 | <0.001 |
| Dorsopathies | 1.95 | 1.76-2.16 | <0.001 | <0.001 |  | 2.05 | 1.78-2.36 | <0.001 | <0.001 |
| Contusion with Intact Skin Surface | 1.95 | 1.62-2.35 | <0.001 | <0.001 |  | 2.57 | 2.03-3.25 | <0.001 | <0.001 |
| Other Diseases of Urinary System | 1.93 | 1.69-2.20 | <0.001 | <0.001 |  | 1.70 | 1.40-2.05 | <0.001 | <0.001 |
| Diseases of Esophagus, Stomach, And Duodenum | 1.92 | 1.69-2.17 | <0.001 | <0.001 |  | 1.96 | 1.65-2.32 | <0.001 | <0.001 |
| Nephritis, Nephrotic Syndrome, And Nephrosis | 1.92 | 1.60-2.30 | <0.001 | <0.001 |  | 2.08 | 1.62-2.67 | <0.001 | <0.001 |
| Other Infectious and Parasitic Diseases | 1.88 | 1.08-3.27 | 0.026 | 0.032 |  | 1.43 | 0.59-3.49 | 0.428 | 0.486 |
| Persons with Potential Health Hazards Related to Personal and Family  History | 1.88 | 1.68-2.10 | <0.001 | <0.001 |  | 2.05 | 1.76-2.39 | <0.001 | <0.001 |
| Human Immunodeficiency Virus (HIV) Infection | 1.86 | 0.87-3.96 | 0.109 | 0.126 |  | 3.96 | 1.72-9.14 | 0.001 | 0.002 |
| Diseases of Oral Cavity, Salivary Glands, And Jaws | 1.84 | 1.47-2.29 | <0.001 | <0.001 |  | 1.95 | 1.45-2.62 | <0.001 | <0.001 |
| Persons with A Condition Influencing Their Health Status | 1.78 | 1.56-2.01 | <0.001 | <0.001 |  | 2.06 | 1.73-2.44 | <0.001 | <0.001 |
| Infections of Skin and Subcutaneous Tissue | 1.76 | 1.49-2.08 | <0.001 | <0.001 |  | 1.59 | 1.23-2.04 | <0.001 | 0.001 |
| Chronic Obstructive Pulmonary Disease and Allied Conditions | 1.73 | 1.52-1.96 | <0.001 | <0.001 |  | 1.72 | 1.44-2.05 | <0.001 | <0.001 |
| Congenital Anomalies | 1.69 | 1.34-2.13 | <0.001 | <0.001 |  | 1.79 | 1.30-2.46 | <0.001 | 0.001 |
| Rheumatism, Excluding the Back | 1.67 | 1.51-1.84 | <0.001 | <0.001 |  | 1.69 | 1.47-1.95 | <0.001 | <0.001 |
| Neoplasms of Unspecified Nature | 1.63 | 1.23-2.17 | 0.001 | 0.001 |  | 1.26 | 0.81-1.96 | 0.306 | 0.355 |
| Viral Diseases Generally Accompanied by Exanthem | 1.59 | 1.20-2.11 | 0.001 | 0.002 |  | 1.46 | 0.96-2.22 | 0.076 | 0.096 |
| Body Mass Index | 1.57 | 1.12-2.20 | 0.009 | 0.012 |  | 1.72 | 1.10-2.69 | 0.018 | 0.025 |
| Malignant Neoplasm of Genitourinary Organs | 1.55 | 1.20-2.02 | 0.001 | 0.001 |  | 1.66 | 1.15-2.40 | 0.006 | 0.009 |
| Noninfectious Enteritis and Colitis | 1.52 | 1.15-2.01 | 0.004 | 0.005 |  | 1.63 | 1.11-2.40 | 0.013 | 0.019 |
| Diseases of Male Genital Organs | 1.52 | 1.31-1.76 | <0.001 | <0.001 |  | 1.64 | 1.34-2.00 | <0.001 | <0.001 |
| Arthropathies And Related Disorders | 1.52 | 1.37-1.69 | <0.001 | <0.001 |  | 1.50 | 1.30-1.74 | <0.001 | <0.001 |
| Osteopathies, Chondropathies, And Acquired Musculoskeletal  Deformities | 1.50 | 1.29-1.73 | <0.001 | <0.001 |  | 1.41 | 1.15-1.74 | 0.001 | 0.002 |
| Diseases of Other Endocrine Glands | 1.47 | 1.29-1.66 | <0.001 | <0.001 |  | 1.38 | 1.15-1.65 | 0.001 | 0.001 |
| Sprains and Strains of Joints and Adjacent Muscles | 1.45 | 1.27-1.66 | <0.001 | <0.001 |  | 1.54 | 1.28-1.86 | <0.001 | <0.001 |
| Malignant Neoplasm of Bone, Connective Tissue, Skin, And Breast | 1.44 | 1.12-1.85 | 0.005 | 0.007 |  | 0.96 | 0.63-1.45 | 0.839 | 0.882 |
| Ischemic Heart Disease | 1.42 | 1.20-1.68 | <0.001 | <0.001 |  | 1.43 | 1.14-1.80 | 0.002 | 0.004 |
| Dislocation | 1.42 | 1.07-1.88 | 0.016 | 0.020 |  | 1.34 | 0.89-2.02 | 0.167 | 0.200 |
| Hernia of Abdominal Cavity | 1.41 | 1.08-1.83 | 0.011 | 0.014 |  | 1.52 | 1.06-2.19 | 0.024 | 0.032 |
| Superficial Injury | 1.40 | 1.09-1.80 | 0.009 | 0.012 |  | 2.32 | 1.75-3.08 | <0.001 | <0.001 |
| Hypertensive Disease | 1.39 | 1.25-1.54 | <0.001 | <0.001 |  | 1.37 | 1.18-1.58 | <0.001 | <0.001 |
| Other Metabolic and Immunity Disorders | 1.38 | 1.25-1.52 | <0.001 | <0.001 |  | 1.38 | 1.20-1.58 | <0.001 | <0.001 |
| Other Diseases Due to Viruses and Chlamydiae | 1.31 | 1.08-1.60 | 0.007 | 0.009 |  | 1.58 | 1.22-2.05 | 0.001 | 0.001 |
| Mycoses | 1.28 | 1.06-1.55 | 0.012 | 0.015 |  | 1.27 | 0.97-1.66 | 0.079 | 0.100 |
| Disorders of Thyroid Gland | 1.27 | 1.07-1.52 | 0.007 | 0.009 |  | 1.63 | 1.30-2.04 | <0.001 | <0.001 |
| Inflammatory Disease of Female Pelvic Organs | 1.26 | 0.87-1.83 | 0.222 | 0.250 |  | 0.94 | 0.52-1.72 | 0.851 | 0.890 |
| Neoplasms of Uncertain Behavior | 1.25 | 0.98-1.61 | 0.075 | 0.090 |  | 1.11 | 0.77-1.59 | 0.582 | 0.653 |
| Persons with Need for Isolation, Other Potential Health Hazards and  Prophylactic Measures | 1.25 | 0.95-1.65 | 0.109 | 0.126 |  | 1.02 | 0.67-1.56 | 0.928 | 0.960 |
| Carcinoma in Situ | 1.23 | 0.69-2.19 | 0.480 | 0.524 |  | 0.83 | 0.34-2.01 | 0.673 | 0.734 |
| Other Diseases of The Upper Respiratory Tract | 1.17 | 1.01-1.35 | 0.036 | 0.045 |  | 1.36 | 1.13-1.64 | 0.001 | 0.002 |
| Other Inflammatory Conditions of Skin and Subcutaneous Tissue | 1.16 | 1.00-1.34 | 0.057 | 0.069 |  | 1.28 | 1.04-1.57 | 0.017 | 0.024 |
| Intestinal Infectious Diseases | 1.14 | 0.70-1.88 | 0.594 | 0.631 |  | 2.02 | 1.18-3.46 | 0.010 | 0.014 |
| Organic Sleep Disorders | 1.14 | 0.87-1.48 | 0.334 | 0.369 |  | 1.42 | 1.01-1.99 | 0.044 | 0.057 |
| Other Diseases of Skin and Subcutaneous Tissue | 1.13 | 1.01-1.27 | 0.033 | 0.041 |  | 1.27 | 1.09-1.49 | 0.003 | 0.004 |
| Diseases of The Ear and Mastoid Process | 1.12 | 0.97-1.30 | 0.109 | 0.126 |  | 1.18 | 0.97-1.44 | 0.091 | 0.112 |
| Other Disorders of Female Genital Tract | 1.07 | 0.87-1.31 | 0.518 | 0.560 |  | 1.29 | 0.99-1.69 | 0.056 | 0.073 |
| Disorders of The Eye and Adnexa | 1.04 | 0.94-1.15 | 0.441 | 0.484 |  | 1.04 | 0.90-1.20 | 0.622 | 0.695 |
| Disorders of Breast | 1.02 | 0.72-1.45 | 0.908 | 0.918 |  | 0.69 | 0.39-1.22 | 0.201 | 0.239 |
| Persons Without Reported Diagnosis Encountered During Examination  and Investigation of Individuals and Populations | 1.00 | 0.91-1.09 | 0.960 | 0.965 |  | 1.03 | 0.90-1.17 | 0.673 | 0.734 |
| Persons with Potential Healthhazards Related to Communicable  Diseases | 0.99 | 0.89-1.10 | 0.835 | 0.856 |  | 1.02 | 0.88-1.19 | 0.759 | 0.811 |
| Acute Respiratory Infections | 0.98 | 0.87-1.11 | 0.781 | 0.809 |  | 0.89 | 0.75-1.06 | 0.203 | 0.240 |
| Benign Neoplasms | 0.78 | 0.65-0.93 | 0.006 | 0.008 |  | 0.82 | 0.64-1.04 | 0.106 | 0.129 |
| Complications Mainly Related to Pregnancy | 0.50 | 0.24-1.05 | 0.069 | 0.082 |  | 0.68 | 0.28-1.65 | 0.393 | 0.449 |
| Persons Encountering Health Services in Circumstances Related to  Reproduction and Development | 0.48 | 0.37-0.64 | <0.001 | 0.000 |  | 0.56 | 0.39-0.81 | 0.002 | 0.003 |
| Injury to Blood Vessels | 0.00 | -Inf-Inf | 0.999 | 0.999 |  | 0.00 | -Inf-Inf | 0.992 | 0.997 |
|  |  |  |  |  |  |  |  |  |  |

Abbreviations: OR, indicates odds ratio; 95% CI, 95% confidence interval; n, number of individuals; FDR: false discovery rate

**Supplemental Table 1B.** Single healthcare indicator odds ratios for death by suicide based on encounter type adjusted by age and sex.

|  | **Discovery**  **(n = 168,206)** | | | |  | **Validation**  **(n = 84,081)** | | | |
| --- | --- | --- | --- | --- | --- | --- | --- | --- | --- |
| **Encounter Type** | **OR** | **95% CI** | **p-value** | **FDR** |  | **OR** | **95% CI** | **p-value** | **FDR** |
| Nonacute Institutional Stay - Rehab | 8.82 | 5.85-13.32 | <0.001 | <0.001 |  | 7.40 | 4.07-13.45 | <0.001 | <0.001 |
| Nonacute Institutional Stay - Other Nonhospital | 6.60 | 2.61-16.69 | <0.001 | <0.001 |  | 0.00 | -Inf-Inf | 1.000 | 1.000 |
| Acute Inpatient - Acute Inpatient | 6.51 | 5.87-7.22 | <0.001 | <0.001 |  | 5.93 | 5.11-6.88 | <0.001 | <0.001 |
| Telephone - Home Health | 4.65 | 2.59-8.33 | <0.001 | <0.001 |  | 2.50 | 0.89-6.97 | 0.081 | 0.102 |
| Other Nonovernight - Skilled Nursing | 4.12 | 2.61-6.50 | <0.001 | <0.001 |  | 3.05 | 1.47-6.30 | 0.003 | 0.004 |
| Nonacute Institutional Stay - Skilled Nursing | 4.08 | 2.98-5.58 | <0.001 | <0.001 |  | 3.32 | 2.11-5.23 | <0.001 | <0.001 |
| Emergency - Hospital Ambulatory | 4.00 | 3.64-4.39 | <0.001 | <0.001 |  | 4.00 | 3.50-4.57 | <0.001 | <0.001 |
| Emergency - Outpatient Clinic | 3.47 | 2.55-4.73 | <0.001 | <0.001 |  | 4.52 | 3.01-6.80 | <0.001 | <0.001 |
| Acute Inpatient - Other Nonhospital | 3.31 | 2.04-5.37 | <0.001 | <0.001 |  | 3.76 | 1.90-7.47 | <0.001 | <0.001 |
| Ambulatory - Observation Bed | 3.30 | 2.59-4.20 | <0.001 | <0.001 |  | 2.25 | 1.51-3.36 | <0.001 | <0.001 |
| Other Nonovernight - Acute Inpatient | 2.63 | 1.15-5.99 | 0.022 | 0.027 |  | 0.00 | -Inf-Inf | 0.973 | 0.989 |
| Nonovernight - Home Health | 2.59 | 2.21-3.02 | <0.001 | <0.001 |  | 2.50 | 2.00-3.13 | <0.001 | <0.001 |
| Ambulatory - Rehab | 2.51 | 2.01-3.14 | <0.001 | <0.001 |  | 2.20 | 1.60-3.02 | <0.001 | <0.001 |
| Emergency - Other Nonhospital | 2.41 | 1.59-3.65 | <0.001 | <0.001 |  | 2.65 | 1.48-4.74 | 0.001 | 0.002 |
| Telephone - Other Nonhospital | 2.25 | 1.97-2.56 | <0.001 | <0.001 |  | 2.18 | 1.80-2.63 | <0.001 | <0.001 |
| Ambulatory - Hospital Ambulatory | 1.85 | 1.66-2.06 | <0.001 | <0.001 |  | 1.85 | 1.59-2.16 | <0.001 | <0.001 |
| Ambulatory - Outpatient Clinic | 1.81 | 1.58-2.07 | <0.001 | <0.001 |  | 1.63 | 1.36-1.97 | <0.001 | <0.001 |
| Nonovernight - Other Nonhospital | 1.80 | 1.57-2.06 | <0.001 | <0.001 |  | 1.44 | 1.17-1.76 | <0.001 | 0.001 |
| Ambulatory - Urgent Care | 1.65 | 1.46-1.87 | <0.001 | <0.001 |  | 1.57 | 1.31-1.87 | <0.001 | <0.001 |
| Lab Only - Outpatient Clinic | 1.56 | 1.38-1.77 | <0.001 | <0.001 |  | 1.71 | 1.43-2.04 | <0.001 | <0.001 |
| Radiology Only - Outpatient Clinic | 1.54 | 1.35-1.75 | <0.001 | <0.001 |  | 1.58 | 1.32-1.90 | <0.001 | <0.001 |
| Ambulatory - Same Day Surgery | 1.52 | 1.33-1.74 | <0.001 | <0.001 |  | 1.71 | 1.42-2.07 | <0.001 | <0.001 |
| Radiology Only - Other Nonhospital | 1.50 | 1.21-1.85 | <0.001 | <0.001 |  | 1.50 | 1.11-2.01 | 0.008 | 0.011 |
| Ambulatory - Other Nonhospital | 1.46 | 1.25-1.71 | <0.001 | <0.001 |  | 1.38 | 1.11-1.73 | 0.004 | 0.006 |
| Email - Other Nonhospital | 1.20 | 1.03-1.40 | 0.016 | 0.020 |  | 1.17 | 0.94-1.45 | 0.157 | 0.190 |
| Lab Only - Other Nonhospital | 1.02 | 0.84-1.25 | 0.807 | 0.831 |  | 1.00 | 0.76-1.33 | 0.986 | 0.996 |
| Other Nonhospital | 0.79 | 0.38-1.62 | 0.515 | 0.559 |  | 0.82 | 0.30-2.29 | 0.708 | 0.765 |
| Nonovernight - Rehab | 0.00 | 0.00-2.09x10^46^ | 0.892 | 0.910 |  | 6.86 | 1.44-32.72 | 0.016 | 0.022 |
|  |  |  |  |  |  |  |  |  |  |

Abbreviations: OR, indicates odds ratio; 95% CI, 95% confidence interval; n, number of individuals; FDR: false discovery rate

**Supplemental Table 1C.** Single healthcare indicator odds ratios for death by suicide based on CPT procedure type adjusted by age and sex.

|  | **Discovery**  **(n = 168,206)** | | | |  | **Validation**  **(n = 84,081)** | | | |
| --- | --- | --- | --- | --- | --- | --- | --- | --- | --- |
| **Procedure Type** | **OR** | **95% CI** | **p-value** | **FDR** |  | **OR** | **95% CI** | **p-value** | **FDR** |
| Critical Care Services | 20.35 | 17.41-23.79 | <0.001 | <0.001 |  | 19.69 | 15.73-24.65 | <0.001 | <0.001 |
| Drug Testing | 13.17 | 11.35-15.29 | <0.001 | <0.001 |  | 15.12 | 12.21-18.71 | <0.001 | <0.001 |
| Psychiatry | 7.92 | 7.17-8.74 | <0.001 | <0.001 |  | 8.04 | 6.99-9.25 | <0.001 | <0.001 |
| Hospital Inpatient Services | 7.71 | 6.85-8.67 | <0.001 | <0.001 |  | 7.61 | 6.44-8.99 | <0.001 | <0.001 |
| Non-Face-To-Face Nonphysician Services | 7.13 | 5.51-9.24 | <0.001 | <0.001 |  | 5.78 | 4.00-8.36 | <0.001 | <0.001 |
| Prolonged Services | 6.78 | 4.33-10.61 | <0.001 | <0.001 |  | 0.00 | 0.00-3.06x10^7^ | 0.641 | 0.708 |
| Therapeutic Drug Assays | 6.59 | 5.69-7.64 | <0.001 | <0.001 |  | 7.36 | 6.01-9.02 | <0.001 | <0.001 |
| Respiratory System | 5.59 | 4.64-6.73 | <0.001 | <0.001 |  | 6.42 | 5.06-8.14 | <0.001 | <0.001 |
| Home Health Services | 5.07 | 2.63-9.79 | <0.001 | <0.001 |  | 7.53 | 2.62-21.65 | <0.001 | <0.001 |
| Radiation Oncology | 4.70 | 3.20-6.92 | <0.001 | <0.001 |  | 3.20 | 1.77-5.77 | <0.001 | <0.001 |
| Emergency Department Services | 4.65 | 4.24-5.11 | <0.001 | <0.001 |  | 5.10 | 4.48-5.80 | <0.001 | <0.001 |
| Qualifying Circumstances for Anesthesia | 4.05 | 3.08-5.33 | <0.001 | <0.001 |  | 3.02 | 1.96-4.66 | <0.001 | <0.001 |
| Non-Face-To-Face Physician Services | 3.95 | 3.16-4.93 | <0.001 | <0.001 |  | 2.09 | 1.46-3.01 | <0.001 | <0.001 |
| Hospital Observation Services | 3.94 | 3.13-4.96 | <0.001 | <0.001 |  | 2.43 | 1.61-3.65 | <0.001 | <0.001 |
| Central Nervous System Assessments/Tests | 3.60 | 2.40-5.39 | <0.001 | <0.001 |  | 2.57 | 1.35-4.88 | 0.004 | 0.006 |
| Moderate | 3.58 | 1.88-6.81 | <0.001 | <0.001 |  | 0.76 | 0.11-5.48 | 0.785 | 0.830 |
| Hydration, Therapeutic, Prophylactic, Diagnostic Injections and  Infusions, And Chemotherapy and Other Highly Complex Drug or  Highly Complex Biologic Agent Administration | 3.45 | 2.99-3.96 | <0.001 | <0.001 |  | 3.20 | 2.62-3.91 | <0.001 | <0.001 |
| Health and Behavior Assessment/Intervention | 3.22 | 1.87-5.55 | <0.001 | <0.001 |  | 7.44 | 4.12-13.42 | <0.001 | <0.001 |
| Cardiovascular | 3.06 | 2.78-3.37 | <0.001 | <0.001 |  | 2.92 | 2.55-3.33 | <0.001 | <0.001 |
| Neurology and Neuromuscular Procedures | 2.79 | 2.29-3.40 | <0.001 | <0.001 |  | 2.22 | 1.63-3.04 | <0.001 | <0.001 |
| Transfusion Medicine | 2.66 | 2.16-3.28 | <0.001 | <0.001 |  | 3.33 | 2.54-4.37 | <0.001 | <0.001 |
| Urinary System | 2.52 | 2.08-3.06 | <0.001 | <0.001 |  | 2.96 | 2.29-3.83 | <0.001 | <0.001 |
| Dialysis | 2.50 | 1.28-4.92 | 0.008 | 0.010 |  | 4.92 | 2.24-10.80 | <0.001 | <0.001 |
| Nursing Facility Services | 2.49 | 1.78-3.46 | <0.001 | <0.001 |  | 3.04 | 1.99-4.66 | <0.001 | <0.001 |
| Noninvasive Vascular Diagnostic Studies | 2.35 | 1.97-2.80 | <0.001 | <0.001 |  | 2.36 | 1.84-3.04 | <0.001 | <0.001 |
| Other Procedures | 2.32 | 1.63-3.30 | <0.001 | <0.001 |  | 2.90 | 1.83-4.58 | <0.001 | <0.001 |
| Pulmonary | 2.30 | 2.01-2.64 | <0.001 | <0.001 |  | 2.60 | 2.17-3.13 | <0.001 | <0.001 |
| Hematology and Coagulation | 2.25 | 2.06-2.47 | <0.001 | <0.001 |  | 2.37 | 2.09-2.70 | <0.001 | <0.001 |
| Consultations | 2.22 | 1.99-2.48 | <0.001 | <0.001 |  | 2.08 | 1.78-2.44 | <0.001 | <0.001 |
| Nuclear Medicine | 2.14 | 1.73-2.64 | <0.001 | <0.001 |  | 1.74 | 1.26-2.41 | 0.001 | 0.001 |
| Radiologic Guidance | 2.14 | 1.57-2.92 | <0.001 | <0.001 |  | 1.50 | 0.89-2.52 | 0.129 | 0.157 |
| Diagnostic Radiology | 2.13 | 1.95-2.32 | <0.001 | <0.001 |  | 2.52 | 2.23-2.86 | <0.001 | <0.001 |
| Hemic and Lymphatic Systems | 1.98 | 0.73-5.40 | 0.183 | 0.209 |  | 4.71 | 2.02-10.95 | <0.001 | 0.001 |
| Special Services, Procedures and Reports | 1.96 | 1.73-2.22 | <0.001 | <0.001 |  | 1.58 | 1.31-1.90 | <0.001 | <0.001 |
| Nervous System | 1.90 | 1.41-2.56 | <0.001 | <0.001 |  | 1.73 | 1.10-2.72 | 0.017 | 0.024 |
| Cardiovascular System | 1.87 | 1.67-2.09 | <0.001 | <0.001 |  | 1.91 | 1.63-2.23 | <0.001 | <0.001 |
| Chemistry | 1.82 | 1.65-2.00 | <0.001 | <0.001 |  | 1.80 | 1.57-2.06 | <0.001 | <0.001 |
| Urinalysis | 1.82 | 1.66-2.00 | <0.001 | <0.001 |  | 1.82 | 1.59-2.08 | <0.001 | <0.001 |
| Diagnostic Ultrasound | 1.78 | 1.57-2.03 | <0.001 | <0.001 |  | 1.81 | 1.51-2.18 | <0.001 | <0.001 |
| Office/Other Outpatient Services | 1.74 | 1.56-1.95 | <0.001 | <0.001 |  | 1.66 | 1.42-1.95 | <0.001 | <0.001 |
| Organ or Disease-Oriented Panels | 1.71 | 1.56-1.88 | <0.001 | <0.001 |  | 1.71 | 1.50-1.95 | <0.001 | <0.001 |
| Immunology | 1.70 | 1.51-1.91 | <0.001 | <0.001 |  | 1.72 | 1.46-2.03 | <0.001 | <0.001 |
| Microbiology | 1.66 | 1.50-1.83 | <0.001 | <0.001 |  | 1.65 | 1.43-1.90 | <0.001 | <0.001 |
| Patient History | 1.66 | 1.03-2.69 | 0.038 | 0.046 |  | 1.12 | 0.52-2.43 | 0.766 | 0.814 |
| Physical Examination | 1.64 | 0.95-2.84 | 0.077 | 0.091 |  | 0.46 | 0.11-1.89 | 0.282 | 0.331 |
| Musculoskeletal System | 1.51 | 1.28-1.79 | <0.001 | <0.001 |  | 1.52 | 1.20-1.93 | <0.001 | 0.001 |
| Acupuncture | 1.48 | 0.81-2.70 | 0.205 | 0.233 |  | 1.53 | 0.67-3.45 | 0.311 | 0.359 |
| Digestive System | 1.47 | 1.27-1.70 | <0.001 | <0.001 |  | 1.22 | 0.98-1.52 | 0.082 | 0.102 |
| Physical Medicine and Rehabilitation | 1.46 | 1.28-1.66 | <0.001 | <0.001 |  | 1.59 | 1.33-1.90 | <0.001 | <0.001 |
| Other Services and Procedures | 1.45 | 1.24-1.70 | <0.001 | <0.001 |  | 1.39 | 1.11-1.74 | 0.005 | 0.007 |
| Special Otorhinolaryngologic Services | 1.42 | 1.15-1.75 | 0.001 | 0.001 |  | 1.23 | 0.90-1.68 | 0.192 | 0.230 |
| Surgical Pathology | 1.33 | 1.17-1.52 | <0.001 | <0.001 |  | 1.30 | 1.08-1.57 | 0.006 | 0.009 |
| Eye and Ocular Adnexa | 1.32 | 0.94-1.86 | 0.108 | 0.126 |  | 1.56 | 1.02-2.38 | 0.042 | 0.055 |
| Integumentary System | 1.31 | 1.16-1.48 | <0.001 | <0.001 |  | 1.39 | 1.17-1.65 | <0.001 | <0.001 |
| Auditory System | 1.18 | 0.84-1.66 | 0.328 | 0.364 |  | 1.53 | 1.00-2.33 | 0.050 | 0.065 |
| Medical Nutrition Therapy | 1.07 | 0.71-1.62 | 0.744 | 0.779 |  | 1.01 | 0.55-1.84 | 0.974 | 0.989 |
| Bone/Joint Studies | 1.06 | 0.71-1.59 | 0.772 | 0.804 |  | 0.99 | 0.57-1.73 | 0.973 | 0.989 |
| Vaccines, Toxoids | 1.03 | 0.93-1.15 | 0.553 | 0.591 |  | 1.01 | 0.87-1.17 | 0.931 | 0.960 |
| Chiropractic Manipulative Treatment | 1.02 | 0.80-1.29 | 0.899 | 0.912 |  | 1.38 | 1.03-1.84 | 0.029 | 0.038 |
| Ophthalmology | 0.98 | 0.88-1.09 | 0.730 | 0.768 |  | 0.97 | 0.83-1.13 | 0.676 | 0.734 |
| Immunization Administration for Vaccines/Toxoids | 0.92 | 0.81-1.04 | 0.178 | 0.204 |  | 1.04 | 0.88-1.23 | 0.631 | 0.700 |
| Allergy and Clinical Immunology | 0.91 | 0.56-1.47 | 0.693 | 0.733 |  | 1.86 | 1.16-2.98 | 0.010 | 0.015 |
| Cytopathology | 0.90 | 0.75-1.10 | 0.303 | 0.339 |  | 1.26 | 0.99-1.60 | 0.062 | 0.079 |
| Other Evaluation and Management Services | 0.75 | 0.31-1.83 | 0.532 | 0.572 |  | 1.60 | 0.65-3.95 | 0.305 | 0.355 |
| Male Genital System | 0.70 | 0.37-1.31 | 0.263 | 0.295 |  | 1.88 | 1.08-3.30 | 0.027 | 0.035 |
| Preventive Medicine Services | 0.65 | 0.58-0.73 | <0.001 | <0.001 |  | 0.78 | 0.67-0.92 | 0.002 | 0.004 |
| Breast Mammography | 0.57 | 0.44-0.75 | <0.001 | <0.001 |  | 0.86 | 0.63-1.18 | 0.357 | 0.409 |
|  |  |  |  |  |  |  |  |  |  |

Abbreviations: OR, indicates odds ratio; 95% CI, 95% confidence interval; n, number of individuals; FDR: false discovery rate

**Supplemental Table 2A.** Multi healthcare indicator models of death by suicide based for individuals with MH diagnosis.

|  | **Conditional Logistic Regression** | | | | | |
| --- | --- | --- | --- | --- | --- | --- |
|  | **Penalized*** | |  | **Standard** | | |
|  | **Discovery OR** | **Validation OR** |  | **OR** | **95% CI** | **p-value** |
| **Diagnosis Sub-Chapter** |  |  |  |  |  |  |
| Poisoning by Drugs, Medicinal and Biological Substances | 3.03 | 2.41 |  | 3.15 | 2.33-4.26 | <0.001 |
| Other Psychoses | 2.89 | 2.81 |  | 4.00 | 3.50-4.58 | <0.001 |
| Neurotic Disorders, Personality Disorders, And Other Nonpsychotic Mental  Disorders | 1.56 | 1.66 |  | 3.58 | 3.00-4.29 | <0.001 |
| Other Disorders of The Central Nervous System | 1.40 | 1.15 |  | 1.44 | 1.20-1.72 | <0.001 |
| Nonspecific Abnormal Findings | 1.20 | - |  | - | - | - |
| Persons Encountering Health Services in Other Circumstances | 1.18 | 1.20 |  | 1.20 | 1.05-1.38 | 0.008 |
| Malignant Neoplasm of Other and Unspecified Sites | 1.17 | - |  | - | - | - |
| Malignant Neoplasm of Bone, Connective Tissue, Skin, And Breast | 1.14 | - |  | - | - | - |
| Other Diseases of Digestive System | 1.13 | - |  | - | - | - |
| Diseases of Male Genital Organs | 1.12 | 1.09 |  | 1.14 | 0.94-1.38 | 0.179 |
| Pain | 1.11 | - |  |  |  |  |
| Symptoms | 1.07 | 1.12 |  | 1.21 | 1.05-1.40 | 0.007 |
| Open Wound of Head, Neck, And Trunk | 1.07 | 1.03 |  | 1.30 | 1.00-1.70 | 0.052 |
| Rheumatism, Excluding the Back | 1.05 | - |  | - | - | - |
| Other and Unspecified Effects of External Causes | 1.03 | - |  | - | - | - |
| Open Wound of Upper Limb | 1.03 | 1.63 |  | 1.53 | 1.15-2.03 | 0.003 |
| Intracranial Injury, Excluding Those with Skull Fracture | 1.03 | - |  |  |  |  |
| Disorders of The Peripheral Nervous System | 1.02 | 0.99 |  | 0.99 | 0.82-1.21 | 0.957 |
| Sprains and Strains of Joints and Adjacent Muscles | 1.02 | - |  | - | - | - |
| Viral Diseases Generally Accompanied by Exanthem | 1.01 | - |  | - | - | - |
| Persons Encountering Health Services for Specific Procedures and Aftercare | 1.00 | 0.87 |  | 0.94 | 0.81-1.10 | 0.444 |
| Other Diseases of Skin and Subcutaneous Tissue | 0.98 | - |  | - | - | - |
| Complications Mainly Related to Pregnancy | 0.98 | - |  | - | - | - |
| Persons with Potential Healthhazards Related to Communicable Diseases | 0.98 | - |  | - | - | - |
| Persons Without Reported Diagnosis Encountered During Examination and  Investigation of Individuals and Populations | 0.97 | 0.77 |  | 0.90 | 0.78-1.02 | 0.107 |
| Disorders of Thyroid Gland | 0.97 | 1.01 |  | 1.03 | 0.84-1.26 | 0.809 |
| Hypertensive Disease | 0.95 | 0.97 |  | 0.93 | 0.81-1.07 | 0.314 |
| Other Inflammatory Conditions of Skin and Subcutaneous Tissue | 0.94 | - |  | - | - | - |
| Diseases of Pulmonary Circulation | 0.94 | 0.92 |  | 0.56 | 0.36-0.88 | 0.011 |
| Intestinal Infectious Diseases | 0.93 | - |  | - | - | - |
| Cerebrovascular Disease | 0.92 | 0.85 |  | 0.68 | 0.53-0.89 | 0.004 |
| Diseases of The Ear and Mastoid Process | 0.91 | - |  | - | - | - |
| Superficial Injury | 0.90 | - |  | - | - | - |
| Disorders of The Eye and Adnexa | 0.88 | 0.93 |  | 0.87 | 0.77-0.99 | 0.039 |
| Ischemic Heart Disease | 0.88 | 0.61 |  | 0.69 | 0.56-0.87 | 0.001 |
| Other Diseases of Urinary System | 0.88 | - |  | - | - | - |
| Nephritis, Nephrotic Syndrome, And Nephrosis | 0.87 | - |  | - | - | - |
| Persons with Potential Health Hazards Related to Personal and Family History | 0.84 | - |  | - | - | - |
| Diseases of The Blood and Blood-Forming Organs | 0.84 | 1.01 |  | 0.91 | 0.75-1.10 | 0.316 |
| Organic Psychotic Conditions | 0.83 | - |  | - | - | - |
| Persons with A Condition Influencing Their Health Status | 0.82 | 1.01 |  | 0.89 | 0.75-1.06 | 0.202 |
| Acute Respiratory Infections | 0.77 | 0.68 |  | 0.65 | 0.56-0.75 | <0.001 |
| Persons Encountering Health Services in Circumstances Related to Reproduction  and Development | 0.75 | 0.73 |  | 0.60 | 0.45-0.81 | 0.001 |
| Other Metabolic and Immunity Disorders | 0.73 | 0.86 |  | 0.73 | 0.64-0.84 | <0.001 |
| Benign Neoplasms | 0.71 | 0.86 |  | 0.68 | 0.55-0.84 | <0.001 |
| Organic Sleep Disorders | 0.66 | - |  | - | - | - |
| **Encounter Type** |  |  |  |  |  |  |
| Acute Inpatient - Acute Inpatient | 1.63 | 1.26 |  | 1.47 | 1.21-1.79 | <0.001 |
| Telephone - Other Nonhospital | 1.41 | - |  | - | - | - |
| Ambulatory - Rehab | 1.36 | 1.07 |  | 1.53 | 1.16-2.02 | 0.003 |
| Emergency - Hospital Ambulatory | 1.09 | - |  | - | - | - |
| Other Nonovernight - Other Nonhospital | 1.09 | 0.89 |  | 0.95 | 0.79-1.13 | 0.547 |
| Email - Other Nonhospital | 1.00 | - |  | - | - | - |
| Ambulatory - Hospital Ambulatory | 0.97 | - |  | - | - | - |
| Ambulatory - Other Nonhospital | 0.97 | 0.95 |  | 0.88 | 0.72-1.07 | 0.202 |
| Other Nonovernight - Home Health | 0.96 | 0.88 |  | 0.87 | 0.71-1.08 | 0.220 |
| Radiology Only - Outpatient Clinic | 0.95 | 0.96 |  | 0.91 | 0.76-1.08 | 0.264 |
| Lab Only - Other Nonhospital | 0.84 | - |  | - | - | - |
| **Procedure Type** |  |  |  |  |  |  |
| Critical Care Services | 3.52 | 3.67 |  | 4.12 | 3.18-5.33 | <0.001 |
| Drug Testing | 2.11 | 2.24 |  | 2.02 | 1.66-2.47 | <0.001 |
| Respiratory System | 1.73 | 1.74 |  | 1.95 | 1.50-2.52 | <0.001 |
| Emergency Department Services | 1.70 | 2.25 |  | 2.05 | 1.78-2.36 | <0.001 |
| Psychiatry | 1.38 | 1.31 |  | 1.24 | 1.08-1.43 | 0.002 |
| Non-Face-To-Face Nonphysician Services | 1.21 | 1.14 |  | 1.53 | 1.13-2.07 | 0.006 |
| Non-Face-To-Face Physician Services | 1.20 | 0.97 |  | 1.13 | 0.86-1.49 | 0.372 |
| Hospital Inpatient Services | 1.15 | 1.26 |  | 1.24 | 1.00-1.55 | 0.051 |
| Cardiovascular | 1.14 | - |  | - | - | - |
| Qualifying Circumstances for Anesthesia | 1.10 | - |  | - | - | - |
| Therapeutic Drug Assays | 1.07 | 1.14 |  | 1.32 | 1.08-1.62 | 0.008 |
| Hematology and Coagulation | 1.07 | 1.25 |  | 1.25 | 1.09-1.44 | 0.002 |
| Special Services, Procedures and Reports | 1.05 | 0.98 |  | 1.09 | 0.92-1.28 | 0.305 |
| Musculoskeletal System | 1.02 | - |  | - | - | - |
| Ophthalmology | 1.02 | - |  | - | - | - |
| Digestive System | 1.01 | - |  | - | - | - |
| Urinary System | 1.01 | 1.13 |  | 1.35 | 1.04-1.76 | 0.024 |
| Hydration, Therapeutic, Prophylactic, Diagnostic Injections and Infusions, And  Chemotherapy and Other Highly Complex Drug or Highly Complex Biologic  Agent Administration | 1.00 | 0.98 |  | 0.95 | 0.79-1.15 | 0.588 |
| Chemistry | 0.97 | - |  | - | - | - |
| Vaccines, Toxoids | 0.97 | 0.84 |  | 0.90 | 0.78-1.03 | 0.115 |
| Microbiology | 0.96 | - |  | - | - | - |
| Other Services and Procedures | 0.93 | - |  | - | - | - |
| Urinalysis | 0.93 | 0.93 |  | 0.83 | 0.72-0.95 | 0.006 |
| Diagnostic Radiology | 0.93 | 1.03 |  | 1.01 | 0.87-1.17 | 0.899 |
| Physical Medicine and Rehabilitation | 0.92 | 1.03 |  | 0.91 | 0.76-1.08 | 0.290 |
| Cytopathology | 0.90 | - |  | - | - | - |
| Preventive Medicine Services | 0.85 | 1.06 |  | 0.93 | 0.80-1.09 | 0.378 |
| Medical Nutrition Therapy | 0.81 | - |  | - | - | - |
| Nursing Facility Services | 0.76 | 0.95 |  | 0.54 | 0.36-0.81 | 0.003 |
| Breast Mammography | 0.68 | - |  | - | - | - |
|  |  |  |  |  |  |  |

All models were adjusted by age and sex

Abbreviations: OR, indicates odds ratio; 95% CI, 95% confidence interval

*Penalized logistic regression using the least absolute shrinkage and selection operator (LASSO)

**Supplemental Table 2B.** Multi healthcare indicator models of death by suicide based for individuals without MH diagnosis.

|  | **Conditional Logistic Regression** | | | | | |
| --- | --- | --- | --- | --- | --- | --- |
|  | **Penalized*** | |  | **Standard** | | |
|  | **Discovery OR** | **Validation OR** |  | **OR** | **95% CI** | **p-value** |
| **Diagnosis Sub-Chapter** |  |  |  |  |  |  |
| Poisoning by Drugs, Medicinal and Biological Substances | 3.36 | - |  | - | - | - |
| Malignant Neoplasm of Other and Unspecified Sites | 3.26 | 4.48 |  | 4.29 | 2.95-6.23 | <0.001 |
| Malignant Neoplasm of Respiratory and Intrathoracic Organs | 2.22 | 1.16 |  | 2.90 | 1.66-5.06 | <0.001 |
| Pain | 1.79 | 1.55 |  | 1.63 | 1.16-2.29 | 0.005 |
| Other Disorders of The Central Nervous System | 1.52 | 1.42 |  | 1.46 | 1.10-1.95 | 0.010 |
| Fracture of Neck and Trunk | 1.44 | - |  | - | - | - |
| Ill-Defined and Unknown Causes of Morbidity and Mortality | 1.43 | - |  | - | - | - |
| Hereditary and Degenerative Diseases of The Central Nervous System | 1.31 | 1.05 |  | 1.37 | 0.97-1.93 | 0.071 |
| Neoplasms of Unspecified Nature | 1.22 | - |  | - | - | - |
| Disorders of The Peripheral Nervous System | 1.22 | 1.03 |  | 1.22 | 0.94-1.59 | 0.131 |
| Diseases of Other Endocrine Glands | 1.20 | - |  | - | - | - |
| Chronic Obstructive Pulmonary Disease and Allied Conditions | 1.20 | 0.93 |  | 0.93 | 0.74-1.16 | 0.513 |
| Other Diseases of Urinary System | 1.18 | 0.72 |  | 0.83 | 0.65-1.06 | 0.140 |
| Burns | 1.15 | - |  | - | - | - |
| Dorsopathies | 1.15 | 1.15 |  | 1.24 | 1.04-1.47 | 0.016 |
| Open Wound of Head, Neck, And Trunk | 1.11 | 1.41 |  | 1.54 | 1.08-2.19 | 0.017 |
| Persons Encountering Health Services for Specific Procedures and Aftercare | 1.09 | - |  | - | - | - |
| Symptoms | 1.08 | 1.07 |  | 1.19 | 1.03-1.38 | 0.021 |
| Fracture of Skull | 1.08 | - |  | - | - | - |
| Other Diseases of Intestines and Peritoneum | 1.07 | - |  | - | - | - |
| Other Diseases of Respiratory System | 1.05 | 1.41 |  | 1.15 | 0.85-1.54 | 0.365 |
| Diseases of The Blood and Blood-Forming Organs | 1.05 | 1.31 |  | 1.01 | 0.78-1.31 | 0.930 |
| Diseases of Male Genital Organs | 1.02 | - |  | - | - | - |
| Diseases of Oral Cavity, Salivary Glands, And Jaws | 1.02 | - |  | - | - | - |
| Nutritional Deficiencies | 1.02 | - |  | - | - | - |
| Osteopathies, Chondropathies, And Acquired Musculoskeletal Deformities | 1.02 | 0.87 |  | 1.02 | 0.80-1.30 | 0.872 |
| Intracranial Injury, Excluding Those with Skull Fracture | 1.01 | - |  | - | - | - |
| Mycoses | 1.00 | - |  | - | - | - |
| Nonspecific Abnormal Findings | 0.99 | 0.69 |  | 0.84 | 0.69-1.01 | 0.066 |
| Superficial Injury | 0.99 | 1.05 |  | 0.93 | 0.63-1.38 | 0.723 |
| Other Diseases Due to Viruses and Chlamydiae | 0.98 | - |  | - | - | - |
| Diseases of Esophagus, Stomach, And Duodenum | 0.97 | 0.87 |  | 0.85 | 0.68-1.07 | 0.158 |
| Sprains and Strains of Joints and Adjacent Muscles | 0.97 | 1.12 |  | 1.05 | 0.84-1.32 | 0.645 |
| Diseases of The Ear and Mastoid Process | 0.96 | 0.96 |  | 0.84 | 0.67-1.04 | 0.112 |
| Persons with Potential Health Hazards Related to Personal and Family History | 0.95 | - |  | - | - | - |
| Other and Unspecified Effects of External Causes | 0.93 | - |  | - | - | - |
| Rheumatism, Excluding the Back | 0.93 | - |  | - | - | - |
| Organic Sleep Disorders | 0.92 | - |  | - | - | - |
| Other Metabolic and Immunity Disorders | 0.92 | 0.73 |  | 0.74 | 0.63-0.87 | <0.001 |
| Hypertensive Disease | 0.91 | - |  | - | - | - |
| Contusion with Intact Skin Surface | 0.91 | - |  | - | - | - |
| Other Forms of Heart Disease | 0.88 | - |  | - | - | - |
| Diseases of Pulmonary Circulation | 0.87 | - |  | - | - | - |
| Noninfectious Enteritis and Colitis | 0.87 | - |  | - | - | - |
| Body Mass Index | 0.86 | - |  | - | - | - |
| Arthropathies And Related Disorders | 0.85 | 0.93 |  | 0.87 | 0.73-1.03 | 0.109 |
| Pneumonia and Influenza | 0.78 | - |  | - | - | - |
| Benign Neoplasms | 0.77 | 0.80 |  | 0.65 | 0.50-0.85 | 0.002 |
| Malignant Neoplasm of Bone, Connective Tissue, Skin, And Breast | 0.77 | - |  | - | - | - |
| Other Diseases of The Upper Respiratory Tract | 0.73 | 1.03 |  | 0.89 | 0.71-1.13 | 0.339 |
| Ischemic Heart Disease | 0.73 | - |  | - | - | - |
| Persons Without Reported Diagnosis Encountered During Examination and  Investigation of Individuals and Populations | 0.72 | - |  | - | - | - |
| Acute Respiratory Infections | 0.71 | 0.61 |  | 0.69 | 0.56-0.84 | <0.001 |
| Other Disorders of Female Genital Tract | 0.68 | - |  | - | - | - |
| Hernia of Abdominal Cavity | 0.61 | - |  | - | - | - |
| Persons Encountering Health Services in Circumstances Related to Reproduction  and Development | 0.37 | 0.66 |  | 0.37 | 0.24-0.58 | <0.001 |
| **Encounter Type** |  |  |  |  |  |  |
| Other Nonovernight - Home Health | 1.44 | 1.39 |  | 1.53 | 1.16-2.00 | 0.002 |
| Radiology Only - Other Non-Hospital | 1.26 | 1.01 |  | 1.54 | 1.14-2.08 | 0.005 |
| Acute Inpatient - Acute Inpatient | 1.16 | - |  | - | - | - |
| Ambulatory - Urgent Care | 1.05 | 1.08 |  | 1.22 | 1.00-1.51 | 0.055 |
| Lab Only - Other Nonhospital | 1.02 | 0.83 |  | 0.91 | 0.72-1.15 | 0.428 |
| Ambulatory - Outpatient Clinic | 1.02 | 0.96 |  | 1.06 | 0.87-1.28 | 0.572 |
| Lab Only - Outpatient Clinic | 0.96 | 0.91 |  | 0.87 | 0.72-1.05 | 0.149 |
| Other Nonhospital | 0.95 | - |  | - | - | - |
| Ambulatory - Other Nonhospital | 0.93 | - |  | - | - | - |
| Other Nonovernight - Other Nonhospital | 0.92 | - |  | - | - | - |
| Emergency - Other Nonhospital | 0.84 | 0.67 |  | 0.68 | 0.52-0.88 | 0.003 |
| Ambulatory - Observation Bed | 0.82 | - |  | - | - | - |
| Emergency - Hospital Ambulatory | 0.63 | 0.68 |  | 0.53 | 0.40-0.70 | <0.001 |
| **Procedure Type** |  |  |  |  |  |  |
| Critical Care Services | 9.90 | 6.64 |  | 9.44 | 6.92-12.88 | <0.001 |
| Emergency Department Services | 2.04 | 1.98 |  | 2.24 | 1.72-2.90 | <0.001 |
| Drug Testing | 1.93 | 2.10 |  | 2.80 | 1.96-4.00 | <0.001 |
| Radiation Oncology | 1.55 | - |  | - | - | - |
| Urinary System | 1.42 | 1.43 |  | 1.54 | 1.12-2.11 | 0.008 |
| Non-Face-To-Face Physician Services | 1.36 | - |  | - | - | - |
| Cardiovascular | 1.21 | 1.13 |  | 1.16 | 0.97-1.38 | 0.095 |
| Special Services, Procedures and Reports | 1.17 | 0.94 |  | 0.99 | 0.81-1.21 | 0.896 |
| Hydration, Therapeutic, Prophylactic, Diagnostic Injections and Infusions, And  Chemotherapy and Other Highly Complex Drug or Highly Complex Biologic  Agent Administration | 1.09 | - |  | - | - | - |
| Eye and Ocular Adnexa | 1.09 | 2.06 |  | 1.89 | 1.23-2.92 | 0.004 |
| Consultations | 1.08 | - |  | - | - | - |
| Digestive System | 1.07 | - |  | - | - | - |
| Pulmonary | 1.06 | 1.03 |  | 1.11 | 0.87-1.41 | 0.417 |
| Hematology and Coagulation | 1.06 | - |  | - | - | - |
| Microbiology | 1.04 | 1.31 |  | 1.15 | 0.97-1.36 | 0.111 |
| Therapeutic Drug Assays | 1.04 | - |  | - | - | - |
| Vaccines, Toxoids | 1.03 | - |  | - | - | - |
| Noninvasive Vascular Diagnostic Studies | 1.03 | 0.93 |  | 1.01 | 0.74-1.37 | 0.957 |
| Other Services and Procedures | 1.01 | - |  | - | - | - |
| Office/Other Outpatient Services | 0.98 | 0.89 |  | 0.89 | 0.74-1.07 | 0.214 |
| Physical Medicine and Rehabilitation | 0.97 | 0.92 |  | 0.81 | 0.65-1.02 | 0.074 |
| Integumentary System | 0.92 | 1.11 |  | 0.98 | 0.81-1.19 | 0.831 |
| Cytopathology | 0.90 | 1.06 |  | 0.96 | 0.71-1.30 | 0.794 |
| Preventive Medicine Services | 0.89 | 0.69 |  | 0.74 | 0.62-0.88 | 0.001 |
| Ophthalmology | 0.87 | 0.89 |  | 0.76 | 0.64-0.90 | 0.001 |
| Special Otorhinolaryngologic Services | 0.79 | - |  | - | - | - |
| Radiologic Guidance | 0.78 | - |  | - | - | - |
| Breast Mammography | 0.70 | 0.85 |  | 0.51 | 0.33-0.79 | 0.003 |
| Immunization Administration for Vaccines/Toxoids | 0.69 | 1.04 |  | 0.82 | 0.68-0.98 | 0.025 |
|  |  |  |  |  |  |  |

All models were adjusted by age and sex

Abbreviations: OR, indicates odds ratio; 95% CI, 95% confidence interval

*Penalized logistic regression using the least absolute shrinkage and selection operator (LASSO)

**Supplemental Table 3A.** Healthcare indicator proportions by LCA group for individuals with MH diagnosis.

|  | **Discovery**  **(n = 23,353)** | | | | | |  | **Validation**  **(n = 11,752)** | | | | | |
| --- | --- | --- | --- | --- | --- | --- | --- | --- | --- | --- | --- | --- | --- |
|  | **Group 1** | **Group 2** | **Group 3** | **Group 4** | **Group 5** | **p-value*** |  | **Group 1** | **Group 2** | **Group 3** | **Group 4** | **Group 5** | **p-value*** |
| Proportion of Sample | 10.0% | 16.1% | 26.0% | 24.1% | 23.8% |  |  | 11.0% | 16.9% | 25.2% | 24.7% | 22.2% |  |
| Proportion of Cases | 25.0% | 31.9% | 19.2% | 15.7% | 8.1% |  |  | 29.0% | 27.8% | 15.8% | 20.4% | 7.0% |  |
| Case Rate (Overall 5.5%) | 13.8% | 11.0% | 4.1% | 3.6% | 1.9% | <0.001 |  | 14.6% | 9.1% | 3.5% | 4.6% | 1.8% | <0.001 |
| **Demographics** |  |  |  |  |  |  |  |  |  |  |  |  |  |
| Age in Years (Mean) | 65.6 | 38.7 | 44.1 | 61.9 | 43.9 | <0.001 |  | 65.1 | 38.5 | 44.4 | 62.0 | 43.6 | <0.001 |
| Sex Male | 46.3 | 30.6 | 53.8 | 42.0 | 29.4 | <0.001 |  | 46.0 | 28.0 | 50.1 | 46.1 | 29.6 | <0.001 |
| **Diagnosis Sub-Chapter** |  |  |  |  |  |  |  |  |  |  |  |  |  |
| Benign Neoplasms | 14.6 | 10.8 | 3.2 | 14.4 | 12.6 | <0.001 |  | 14.7 | 11.5 | 2.8 | 14.1 | 11.9 | <0.001 |
| Disorders of Thyroid Gland | 20.0 | 8.8 | 1.7 | 14.9 | 7.3 | <0.001 |  | 21.7 | 8.0 | 1.6 | 13.7 | 6.9 | <0.001 |
| Other Metabolic and Immunity Disorders | 80.6 | 26.2 | 8.4 | 58.5 | 23.2 | <0.001 |  | 82.0 | 22.4 | 6.6 | 60.5 | 20.7 | <0.001 |
| Diseases of The Blood and Blood-Forming Organs | 47.4 | 11.2 | 0.6 | 7.5 | 2.8 | <0.001 |  | 44.9 | 11.0 | 0.3 | 6.7 | 1.9 | <0.001 |
| Other Psychoses | 36.9 | 41.1 | 16.3 | 22.3 | 20.4 | <0.001 |  | 37.4 | 34.7 | 14.9 | 21.6 | 20.9 | <0.001 |
| Neurotic Disorders, Personality Disorders, And Other  Nonpsychotic Mental Disorders | 81.4 | 89.8 | 58.5 | 74.6 | 78.2 | <0.001 |  | 82.1 | 89.5 | 58.7 | 75.0 | 77.3 | <0.001 |
| Other Disorders of The Central Nervous System | 19.3 | 17.9 | 1.8 | 5.1 | 4.5 | <0.001 |  | 19.5 | 16.3 | 2.2 | 5.1 | 3.2 | <0.001 |
| Disorders of The Peripheral Nervous System | 26.1 | 6.1 | 1.2 | 13.3 | 1.7 | <0.001 |  | 24.7 | 5.9 | 0.8 | 11.9 | 1.7 | <0.001 |
| Disorders of The Eye and Adnexa | 54.6 | 23.9 | 13.9 | 48.0 | 26.5 | <0.001 |  | 52.5 | 25.6 | 14.3 | 47.1 | 25.9 | <0.001 |
| Hypertensive Disease | 75.2 | 9.5 | 7.7 | 56.5 | 10.7 | <0.001 |  | 72.8 | 8.7 | 5.8 | 55.8 | 9.9 | <0.001 |
| Ischemic Heart Disease | 33.6 | 0.3 | 0.5 | 11.0 | 0.2 | <0.001 |  | 31.7 | 0.3 | 0.2 | 10.3 | 0.1 | <0.001 |
| Diseases of Pulmonary Circulation | 10.5 | 0.1 | 0.0 | 1.1 | 0.0 | <0.001 |  | 9.0 | 0.5 | 0.1 | 0.7 | 0.0 | <0.001 |
| Cerebrovascular Disease | 24.2 | 1.0 | 0.1 | 5.0 | 0.2 | <0.001 |  | 21.9 | 0.6 | 0.0 | 5.2 | 0.0 | <0.001 |
| Acute Respiratory Infections | 22.9 | 37.1 | 16.4 | 18.4 | 21.4 | <0.001 |  | 20.8 | 37.5 | 17.1 | 17.2 | 21.0 | <0.001 |
| Diseases of Male Genital Organs | 16.0 | 2.2 | 2.4 | 9.6 | 2.4 | <0.001 |  | 14.1 | 2.2 | 2.4 | 10.6 | 2.2 | <0.001 |
| Symptoms | 98.3 | 86.1 | 25.0 | 71.1 | 43.8 | <0.001 |  | 97.7 | 84.7 | 24.4 | 70.2 | 41.5 | <0.001 |
| Open Wound of Head, Neck, And Trunk | 9.4 | 4.3 | 1.1 | 2.1 | 0.4 | <0.001 |  | 9.1 | 4.6 | 1.0 | 1.5 | 0.2 | <0.001 |
| Open Wound of Upper Limb | 4.8 | 3.7 | 0.9 | 2.3 | 0.5 | <0.001 |  | 4.6 | 3.5 | 1.7 | 1.8 | 0.7 | <0.001 |
| Poisoning by Drugs, Medicinal and Biological Substances | 5.4 | 3.5 | 0.1 | 0.0 | 0.0 | <0.001 |  | 6.0 | 2.6 | 0.0 | 0.1 | 0.0 | <0.001 |
| Persons Encountering Health Services in Circumstances Related  to Reproduction and Development | 0.9 | 23.0 | 6.8 | 0.1 | 14.1 | <0.001 |  | 1.6 | 24.3 | 6.5 | 0.1 | 15.4 | <0.001 |
| Persons with A Condition Influencing Their Health Status | 62.7 | 9.6 | 0.9 | 22.9 | 3.6 | <0.001 |  | 59.8 | 10.6 | 0.9 | 21.1 | 2.7 | <0.001 |
| Persons Encountering Health Services for Specific Procedures  and Aftercare | 74.4 | 26.3 | 3.1 | 28.6 | 4.1 | <0.001 |  | 71.9 | 24.9 | 3.2 | 24.8 | 4.2 | <0.001 |
| Persons Encountering Health Services in Other Circumstances | 49.8 | 36.1 | 11.9 | 24.0 | 14.0 | <0.001 |  | 49.6 | 33.4 | 9.4 | 22.5 | 12.3 | <0.001 |
| Persons Without Reported Diagnosis Encountered During  Examination and Investigation of Individuals and Populations | 74.1 | 63.9 | 10.8 | 75.6 | 99.5 | <0.001 |  | 74.1 | 64.6 | 11.4 | 75.1 | 98.6 | <0.001 |
| **Encounter Type** |  |  |  |  |  |  |  |  |  |  |  |  |  |
| Ambulatory - Other Nonhospital | 24.9 | 11.8 | 5.5 | 16.7 | 6.1 | <0.001 |  | 19.7 | 11.7 | 6.0 | 16.2 | 7.7 | <0.001 |
| Ambulatory - Rehab | 9.6 | 11.2 | 2.0 | 3.8 | 3.1 | <0.001 |  | 9.7 | 10.1 | 1.9 | 2.4 | 3.6 | <0.001 |
| Acute Inpatient - Acute Inpatient | 77.0 | 27.4 | 0.7 | 3.3 | 0.2 | <0.001 |  | 75.5 | 23.3 | 1.0 | 3.2 | 0.1 | <0.001 |
| Other Nonovernight - Home Health | 38.0 | 5.4 | 1.2 | 8.8 | 0.4 | <0.001 |  | 35.2 | 5.3 | 0.7 | 7.7 | 0.9 | <0.001 |
| Other Nonovernight - Other Nonhospital | 32.7 | 19.3 | 4.3 | 30.0 | 10.1 | <0.001 |  | 35.4 | 19.1 | 3.6 | 24.1 | 10.8 | <0.001 |
| Radiology Only - Outpatient Clinic | 45.4 | 31.6 | 5.4 | 43.4 | 15.6 | <0.001 |  | 45.9 | 35.3 | 5.7 | 41.5 | 14.8 | <0.001 |
| **Procedure Type** |  |  |  |  |  |  |  |  |  |  |  |  |  |
| Critical Care Services | 16.1 | 2.9 | 0.0 | 0.0 | 0.0 | <0.001 |  | 14.2 | 1.8 | 0.0 | 0.2 | 0.0 | <0.001 |
| Diagnostic Radiology | 98.6 | 72.0 | 18.4 | 65.3 | 30.8 | <0.001 |  | 97.7 | 70.8 | 16.5 | 62.7 | 27.4 | <0.001 |
| Drug Testing | 8.9 | 13.4 | 1.7 | 2.4 | 0.2 | <0.001 |  | 10.8 | 10.8 | 1.2 | 2.4 | 0.9 | <0.001 |
| Emergency Department Services | 71.2 | 54.3 | 6.2 | 14.6 | 3.8 | <0.001 |  | 69.1 | 49.1 | 6.8 | 12.6 | 2.6 | <0.001 |
| Hematology and Coagulation | 92.3 | 79.4 | 12.0 | 64.9 | 38.7 | <0.001 |  | 91.3 | 79.1 | 10.4 | 62.3 | 36.2 | <0.001 |
| Hospital Inpatient Services | 56.3 | 13.0 | 0.1 | 0.3 | 0.0 | <0.001 |  | 52.5 | 9.3 | 0.3 | 0.3 | 0.0 | <0.001 |
| Hydration, Therapeutic, Prophylactic, Diagnostic Injections and  Infusions, And Chemotherapy and Other Highly Complex  Drug or Highly Complex Biologic Agent Administration | 28.4 | 23.9 | 1.0 | 6.7 | 1.3 | <0.001 |  | 29.5 | 23.5 | 0.8 | 6.6 | 1.0 | <0.001 |
| Non-Face-To-Face Nonphysician Services | 7.0 | 6.1 | 0.8 | 2.9 | 1.0 | <0.001 |  | 6.9 | 7.2 | 0.6 | 2.0 | 1.0 | <0.001 |
| Non-Face-To-Face Physician Services | 11.3 | 6.2 | 1.1 | 7.6 | 1.4 | <0.001 |  | 12.5 | 7.6 | 1.4 | 5.4 | 0.9 | <0.001 |
| Nursing Facility Services | 14.0 | 0.0 | 0.1 | 0.9 | 0.0 | <0.001 |  | 14.2 | 0.0 | 0.0 | 0.8 | 0.0 | <0.001 |
| Physical Medicine and Rehabilitation | 32.8 | 22.7 | 5.1 | 19.4 | 8.5 | <0.001 |  | 32.3 | 22.6 | 4.1 | 16.3 | 10.2 | <0.001 |
| Preventive Medicine Services | 15.4 | 27.1 | 1.7 | 25.5 | 72.0 | <0.001 |  | 16.3 | 28.1 | 1.6 | 25.6 | 75.2 | <0.001 |
| Psychiatry | 23.2 | 49.1 | 24.9 | 14.8 | 32.6 | <0.001 |  | 28.7 | 44.1 | 24.0 | 16.6 | 32.4 | <0.001 |
| Respiratory System | 8.9 | 4.2 | 0.5 | 2.4 | 0.7 | <0.001 |  | 8.6 | 3.3 | 0.1 | 2.6 | 1.0 | <0.001 |
| Special Services, Procedures and Reports | 31.9 | 23.5 | 3.6 | 15.1 | 7.0 | <0.001 |  | 28.4 | 22.5 | 2.5 | 14.7 | 8.0 | <0.001 |
| Therapeutic Drug Assays | 14.6 | 9.1 | 0.8 | 2.9 | 0.6 | <0.001 |  | 17.1 | 8.0 | 0.8 | 2.4 | 0.7 | <0.001 |
| Urinalysis | 66.5 | 59.5 | 7.3 | 33.3 | 25.9 | <0.001 |  | 63.2 | 59.2 | 6.1 | 31.2 | 25.1 | <0.001 |
| Urinary System | 12.6 | 2.9 | 0.1 | 4.7 | 0.3 | <0.001 |  | 13.7 | 2.5 | 0.1 | 4.4 | 0.2 | <0.001 |
| Vaccines, Toxoids | 46.1 | 27.5 | 13.0 | 49.5 | 30.8 | <0.001 |  | 50.1 | 30.2 | 12.1 | 46.5 | 33.7 | <0.001 |
|  |  |  |  |  |  |  |  |  |  |  |  |  |  |

Abbreviations: n, indicates number of individuals

*ANOVA for continuous variables, chi-squared test for categorical variables

**Supplemental Table 3B.** Healthcare indicator proportions by LCA group for individuals without MH diagnosis.

|  | **Discovery**  **(n = 52,957)** | | | | | |  | **Validation**  **(n = 25,439)** | | | | | |
| --- | --- | --- | --- | --- | --- | --- | --- | --- | --- | --- | --- | --- | --- |
|  | **Group 1** | **Group 2** | **Group 3** | **Group 4** | **Group 5** | **p-value*** |  | **Group 1** | **Group 2** | **Group 3** | **Group 4** | **Group 5** | **p-value*** |
| Proportion of Sample | 14.1% | 21.8% | 25.6% | 25.4% | 13.3% |  |  | 14.2% | 18.3% | 29.9% | 23.6% | 14.1% |  |
| Proportion of Cases | 25.5% | 24.6% | 25.7% | 21.3% | 3.0% |  |  | 22.6% | 22.1% | 33.6% | 17.6% | 4.1% |  |
| Case Rate (Overall 1.5%) | 2.7% | 1.7% | 1.5% | 1.3% | 0.3% | <0.001 |  | 2.4% | 1.8% | 1.7% | 1.1% | 0.4% | <0.001 |
| **Demographics** |  |  |  |  |  |  |  |  |  |  |  |  |  |
| Age in Years (Mean) | 58.7 | 42.9 | 39.9 | 60.9 | 38.7 | <0.001 |  | 57.2 | 42.7 | 41.8 | 61.7 | 38.0 | <0.001 |
| Sex Male | 42.9 | 58.2 | 58.1 | 52.0 | 7.4 | <0.001 |  | 43.6 | 58.7 | 61.3 | 50.0 | 6.5 | <0.001 |
| **Diagnosis Sub-Chapter** |  |  |  |  |  |  |  |  |  |  |  |  |  |
| Malignant Neoplasm of Respiratory and Intrathoracic Organs | 1.1 | 0.0 | 0.0 | 0.2 | 0.0 | <0.001 |  | 1.1 | 0.0 | 0.0 | 0.3 | 0.0 | <0.001 |
| Malignant Neoplasm of Other and Unspecified Sites | 2.7 | 0.0 | 0.1 | 0.7 | 0.2 | <0.001 |  | 2.7 | 0.0 | 0.0 | 0.6 | 0.3 | <0.001 |
| Benign Neoplasms | 15.0 | 0.1 | 3.1 | 11.4 | 9.8 | <0.001 |  | 14.7 | 0.1 | 2.6 | 12.8 | 8.7 | <0.001 |
| Other Metabolic and Immunity Disorders | 57.2 | 0.3 | 3.2 | 48.6 | 12.3 | <0.001 |  | 52.0 | 0.5 | 4.3 | 54.0 | 9.5 | <0.001 |
| Diseases of The Blood and Blood-Forming Organs | 18.8 | 0.0 | 0.4 | 4.2 | 4.8 | <0.001 |  | 17.7 | 0.0 | 0.3 | 5.3 | 5.5 | <0.001 |
| Hereditary and Degenerative Diseases of The Central Nervous  System | 6.2 | 0.0 | 0.2 | 2.2 | 0.2 | <0.001 |  | 5.9 | 0.0 | 0.1 | 2.3 | 0.2 | <0.001 |
| Pain | 7.9 | 0.0 | 0.5 | 1.2 | 0.6 | <0.001 |  | 7.3 | 0.0 | 0.3 | 1.5 | 0.3 | <0.001 |
| Other Disorders of The Central Nervous System | 9.2 | 0.0 | 2.1 | 1.7 | 4.1 | <0.001 |  | 8.6 | 0.0 | 1.8 | 2.3 | 4.1 | <0.001 |
| Disorders of The Peripheral Nervous System | 13.6 | 0.0 | 0.9 | 5.2 | 1.3 | <0.001 |  | 13.5 | 0.0 | 0.5 | 5.4 | 1.9 | <0.001 |
| Diseases of The Ear and Mastoid Process | 17.5 | 0.1 | 9.1 | 9.1 | 6.5 | <0.001 |  | 18.7 | 0.1 | 7.7 | 9.7 | 7.3 | <0.001 |
| Acute Respiratory Infections | 27.0 | 0.1 | 23.8 | 7.1 | 23.9 | <0.001 |  | 29.7 | 0.0 | 17.8 | 8.4 | 25.7 | <0.001 |
| Other Diseases of The Upper Respiratory Tract | 19.0 | 0.1 | 10.3 | 6.0 | 10.5 | <0.001 |  | 20.5 | 0.2 | 7.8 | 6.2 | 10.3 | <0.001 |
| Chronic Obstructive Pulmonary Disease and Allied Conditions | 24.3 | 0.0 | 7.5 | 5.2 | 6.0 | <0.001 |  | 26.5 | 0.0 | 5.8 | 5.4 | 6.0 | <0.001 |
| Other Diseases of Respiratory System | 12.3 | 0.0 | 0.7 | 0.9 | 0.8 | <0.001 |  | 12.6 | 0.0 | 0.6 | 0.9 | 0.3 | <0.001 |
| Diseases of Esophagus, Stomach, And Duodenum | 26.2 | 0.1 | 2.7 | 8.5 | 4.1 | <0.001 |  | 25.4 | 0.0 | 2.0 | 10.0 | 3.8 | <0.001 |
| Other Diseases of Urinary System | 26.0 | 0.0 | 3.1 | 3.6 | 10.3 | <0.001 |  | 24.8 | 0.0 | 1.3 | 4.4 | 12.1 | <0.001 |
| Arthropathies And Related Disorders | 44.9 | 0.1 | 10.6 | 22.2 | 8.4 | <0.001 |  | 44.1 | 0.0 | 8.8 | 25.2 | 8.8 | <0.001 |
| Dorsopathies | 35.4 | 0.1 | 10.0 | 13.4 | 8.3 | <0.001 |  | 35.5 | 0.1 | 7.8 | 15.8 | 8.7 | <0.001 |
| Osteopathies, Chondropathies, And Acquired  Musculoskeletal Deformities | 22.3 | 0.0 | 3.1 | 10.2 | 4.9 | <0.001 |  | 19.6 | 0.0 | 2.5 | 12.4 | 4.0 | <0.001 |
| Symptoms | 90.0 | 0.3 | 28.3 | 34.3 | 38.1 | <0.001 |  | 90.2 | 0.2 | 20.9 | 40.3 | 39.8 | <0.001 |
| Nonspecific Abnormal Findings | 37.4 | 0.2 | 1.5 | 21.3 | 15.1 | <0.001 |  | 34.2 | 0.1 | 1.8 | 23.2 | 15.3 | <0.001 |
| Sprains and Strains of Joints and Adjacent Muscles | 17.1 | 0.0 | 11.6 | 4.6 | 5.4 | <0.001 |  | 17.3 | 0.0 | 9.0 | 5.7 | 6.0 | <0.001 |
| Open Wound of Head, Neck, And Trunk | 4.3 | 0.0 | 1.9 | 0.4 | 0.5 | <0.001 |  | 4.8 | 0.0 | 1.3 | 0.5 | 0.4 | <0.001 |
| Superficial Injury | 4.8 | 0.0 | 2.5 | 1.2 | 1.3 | <0.001 |  | 4.7 | 0.0 | 2.0 | 1.7 | 1.0 | <0.001 |
| Persons Encountering Health Services in Circumstances  Related to Reproduction and Development | 3.5 | 1.0 | 8.3 | 0.1 | 35.8 | <0.001 |  | 4.6 | 0.1 | 5.3 | 0.0 | 35.8 | <0.001 |
| **Encounter Type** |  |  |  |  |  |  |  |  |  |  |  |  |  |
| Ambulatory - Outpatient Clinic | 99.2 | 16.9 | 90.5 | 94.9 | 97.3 | <0.001 |  | 99.4 | 5.1 | 89.8 | 95.0 | 96.6 | <0.001 |
| Ambulatory - Urgent Care | 17.9 | 2.3 | 18.6 | 4.4 | 10.7 | <0.001 |  | 18.0 | 0.7 | 13.0 | 4.2 | 13.5 | <0.001 |
| Emergency - Hospital Ambulatory | 37.3 | 0.1 | 10.5 | 0.0 | 3.3 | <0.001 |  | 41.0 | 0.2 | 7.1 | 0.0 | 4.4 | <0.001 |
| Email - Other Nonhospital | 17.3 | 1.6 | 3.9 | 12.3 | 11.5 | <0.001 |  | 16.3 | 1.4 | 3.5 | 12.0 | 11.6 | <0.001 |
| Lab Only - Outpatient Clinic | 39.8 | 2.8 | 11.9 | 29.0 | 29.8 | <0.001 |  | 38.5 | 2.3 | 10.6 | 28.9 | 29.3 | <0.001 |
| Lab Only - Other Nonhospital | 21.3 | 1.2 | 2.4 | 19.0 | 18.3 | <0.001 |  | 19.9 | 1.0 | 3.1 | 23.8 | 17.2 | <0.001 |
| Other Nonovernight - Home Health | 13.0 | 0.2 | 0.8 | 1.9 | 2.0 | <0.001 |  | 12.5 | 0.2 | 0.6 | 1.8 | 2.3 | <0.001 |
| Radiology Only - Other Nonhospital | 10.2 | 0.5 | 2.4 | 6.2 | 11.1 | <0.001 |  | 9.9 | 0.5 | 1.7 | 6.9 | 10.7 | <0.001 |
| **Procedure Type** |  |  |  |  |  |  |  |  |  |  |  |  |  |
| Breast Mammography | 17.0 | 0.9 | 0.0 | 13.5 | 15.6 | <0.001 |  | 14.0 | 0.7 | 0.2 | 16.4 | 14.6 | <0.001 |
| Cardiovascular | 56.0 | 0.2 | 4.5 | 17.2 | 4.1 | <0.001 |  | 53.6 | 0.1 | 3.9 | 19.9 | 4.6 | <0.001 |
| Critical Care Services | 3.3 | 0.0 | 0.1 | 0.1 | 0.0 | <0.001 |  | 3.0 | 0.0 | 0.1 | 0.1 | 0.0 | <0.001 |
| Cytopathology | 13.4 | 0.4 | 0.0 | 3.6 | 58.2 | <0.001 |  | 12.0 | 0.1 | 0.0 | 5.8 | 53.2 | <0.001 |
| Drug Testing | 3.2 | 0.2 | 1.0 | 0.4 | 0.9 | <0.001 |  | 3.2 | 0.2 | 0.8 | 0.3 | 0.7 | <0.001 |
| Emergency Department Services | 42.2 | 0.1 | 10.4 | 0.1 | 3.6 | <0.001 |  | 45.9 | 0.1 | 6.7 | 0.1 | 4.9 | <0.001 |
| Eye and Ocular Adnexa | 3.1 | 0.0 | 0.0 | 1.8 | 0.0 | <0.001 |  | 3.7 | 0.0 | 0.0 | 2.1 | 0.0 | <0.001 |
| Immunization Administration for Vaccines/Toxoids | 27.7 | 2.5 | 8.8 | 25.1 | 24.3 | <0.001 |  | 25.5 | 0.6 | 10.9 | 26.6 | 21.0 | <0.001 |
| Integumentary System | 22.4 | 0.1 | 6.9 | 14.5 | 7.3 | <0.001 |  | 22.0 | 0.1 | 6.0 | 15.6 | 6.3 | <0.001 |
| Microbiology | 46.2 | 0.4 | 19.0 | 6.3 | 58.7 | <0.001 |  | 45.7 | 0.2 | 12.5 | 8.2 | 61.2 | <0.001 |
| Noninvasive Vascular Diagnostic Studies | 11.4 | 0.0 | 0.3 | 2.5 | 0.7 | <0.001 |  | 10.2 | 0.1 | 0.3 | 3.0 | 0.8 | <0.001 |
| Office/Other Outpatient Services | 98.8 | 2.3 | 89.8 | 87.8 | 80.3 | <0.001 |  | 98.7 | 0.2 | 78.3 | 91.3 | 81.9 | <0.001 |
| Ophthalmology | 35.4 | 6.7 | 10.2 | 30.3 | 17.2 | <0.001 |  | 36.0 | 0.4 | 15.9 | 30.4 | 15.7 | <0.001 |
| Physical Medicine and Rehabilitation | 25.7 | 0.0 | 6.2 | 7.4 | 4.5 | <0.001 |  | 26.3 | 0.0 | 4.7 | 9.4 | 4.6 | <0.001 |
| Preventive Medicine Services | 27.7 | 3.3 | 8.3 | 32.4 | 67.6 | <0.001 |  | 26.0 | 0.2 | 13.3 | 34.8 | 62.9 | <0.001 |
| Pulmonary | 23.2 | 0.0 | 4.6 | 1.0 | 2.2 | <0.001 |  | 23.7 | 0.1 | 3.2 | 1.1 | 2.7 | <0.001 |
| Special Services, Procedures and Reports | 25.6 | 0.5 | 5.9 | 10.3 | 16.3 | <0.001 |  | 26.0 | 0.3 | 4.1 | 10.6 | 15.4 | <0.001 |
| Urinary System | 8.7 | 0.0 | 0.1 | 1.2 | 0.8 | <0.001 |  | 7.5 | 0.0 | 0.0 | 1.7 | 1.0 | <0.001 |
|  |  |  |  |  |  |  |  |  |  |  |  |  |  |

Abbreviations: n, indicates number of individuals

*ANOVA for continuous variables, chi-squared test for categorical variables
