# Supplementary material for: Detecting and distinguishing indicators of risk for suicide using clinical records
Source: Transl Psychiatry. 2022 Jul 13;12:280. doi: 10.1038/s41398-022-02051-4 (PMC9279332; doi:10.1038/s41398-022-02051-4)
Supplement: Supplementary file 2 — Supplementary Figure 1 [file 41398_2022_2051_MOESM2_ESM.docx]

**Supplementary Figure 1: Description of the goals and samples used in the analyses assessing the association between health care indicators and risk of suicide mortality.** For the purposes of analyses, the overall sample was divided into discovery and validation (shaded grey) to assess the reproducibility of findings. Further subdivision into those with a mental health diagnosis (MH) and those without (non-MH) was performed to assess the differential effects in these disparate suicide risk strata. The two goals and the corresponding analyses to achieve them are listed on the left hand side of the figure, and the discovery and validation sample sets for each corresponding analysis are indicated by “x”s in the grid to the right of the analyses and under the study sample tree.
