# Supplementary material for: Detecting and distinguishing indicators of risk for suicide using clinical records
Source: Transl Psychiatry. 2022 Jul 13;12:280. doi: 10.1038/s41398-022-02051-4 (PMC9279332; doi:10.1038/s41398-022-02051-4)

**Supplementary Figure 2.** Bayesian information criterion (BIC) for differing number of LCA groups in the non-mental health and mental health strata and the respective discovery and validation samples.


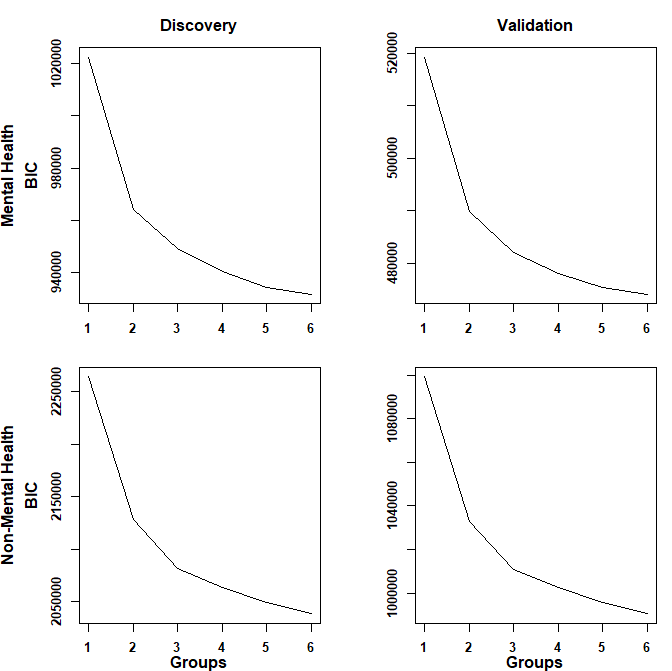

Supplement: Supplementary file 3 — Supplementary Figure 2 [file 41398_2022_2051_MOESM3_ESM.docx]
